# Synthesis, Anticancer Activity, and Preliminary Pharmacokinetic Evaluation of 4,4-Disubstituted Curcuminoid 2,2-bis(Hydroxymethyl)Propionate Derivatives

**DOI:** 10.3390/molecules25030479

**Published:** 2020-01-22

**Authors:** Der-Yen Lee, Yu-Chi Hou, Jai-Sing Yang, Hui-Yi Lin, Tsu-Yuan Chang, Kuo-Hsiung Lee, Sheng-Chu Kuo, Min-Tsang Hsieh

**Affiliations:** 1 Graduate Institute of Integrated Medicine, China Medical University, No. 91, Hsueh-Shih Road, Taichung 40402, Taiwan; deryen.lee@mail.cmu.edu.tw; 2 School of Pharmacy, China Medical University, Taichung 40402, Taiwan; houyc@mail.cmu.edu.tw (Y.-C.H.); u103003309@cmu.edu.tw (T.-Y.C.); 3 Department of Medical Research, China Medical University Hospital, China Medical University, Taichung 40447, Taiwan; jaisingyang@gmail.com; 4 Research Center for Chinese Herbal Medicine, China Medical University, Taichung 404, Taiwan; pingababy@yahoo.com.tw; 5 Natural Products Research Laboratories, UNC Eshelman School of Pharmacy, University of North Carolina, Chapel Hill, NC 27599, USA; khlee@unc.edu; 6 Chinese Medicinal Research and Development Center, China Medical University Hospital, Taichung 40447, Taiwan

**Keywords:** curcuminoid derivatives, prodrug, colon cancer, breast cancer, active metabolites

## Abstract

Compound **1** is a curcumin di-*O*-2,2-bis(hydroxymethyl)propionate that shows significant in vitro and in vivo inhibitory activity against MDA-MB-231 cells with eight to ten-fold higher potency than curcumin. Here, we modified the α-position (C-4 position) of the central 1,3-diketone moiety of **1** with polar or nonpolar functional groups to afford a series of 4,4-disubstituted curcuminoid 2,2-bis(hydroxymethyl)propionate derivatives and evaluated their anticancer activities. A clear structure–activity relationship of compound **1** derivatives focusing on the functional groups at the C-4 position was established based on their anti-proliferative effects against the MDA-MB-231 and HCT-116 cell lines. Compounds **2**–**6** are 4,4-dimethylated, 4,4-diethylated, 4,4-dibenzylated, 4,4-dipropargylated and 4,4-diallylated compound **1**, respectively. Compounds **2m**–**6m**, the ester hydrolysis products of compounds **2**–**6**, respectively, were synthesized and assessed for anticancer activity. Among all compound **1** derivatives, compound **2** emerged as a potential chemotherapeutic agent for colon cancer due to the promising in vivo anti-proliferative activities of **2** (IC_50_ = 3.10 ± 0.29 μM) and its ester hydrolysis product **2m** (IC_50_ = 2.17 ± 0.16 μM) against HCT-116. The preliminary pharmacokinetic evaluation of **2** implied that **2** and **2m** are main contributors to the in vivo efficacy. Compound **2** was further evaluated in an animal study using HCT-116 colon tumor xenograft bearing nude mice. The results revealed a dose-dependent efficacy that led to tumor volume reductions of 27%, 45%, and 60% at 50, 100, and 150 mg/kg doses, respectively. The established structure–activity relationship and pharmacokinetic outcomes of **2** is the guidance for future development of 4,4-disubstituted curcuminoid 2,2-bis(hydroxymethyl)- propionate derivatives as anticancer drug candidates.

## 1. Introduction

**Scheme 1:**
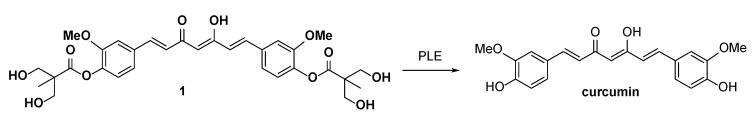
PLE mediated hydrolysis of **1**.

Curcumin [(1*E*,6*E*)-1,7-bis(4-hydroxy-3-methoxyphenyl)hepta-1,6-diene-3,5-dione, the structure of which is shown in Scheme 1], the naturally occurring phytochemical from the rhizome of *Curcuma* *longa* L., is a polyphenol with a symmetrical structure composed of two *ortho*-methoxyphenol rings connected to each other through a flexible conjugated hydrocarbon chain. It is a versatile therapeutic agent against cancer [1] and exhibits diverse pharmacological effects, including antidiabetic [2], antiviral [3], analgesic [4], nephroprotective [5], and cardioprotective effects [6], via regulating the gene expression [7] and protein binding [8]. Similar to some phenolic natural products, curcumin is a multi-target anticancer agent [9] with the ability to interfere with several signaling pathways associated with tumor progression, metastasis, apoptosis, and angiogenesis [10].

While curcumin shows promising therapeutic properties, its low solubility and poor chemical and metabolic stability hinder further drug development [11]. The low chemical stability of curcumin is demonstrated by its rapid decomposition upon exposure to light or high temperatures (>70 °C) [12]. The compound decomposes into several degradation products, including a bicyclopentadione derivative, vanillin, feruloylmethane, and ferulic acid. The degradation of curcumin presumably results from its autoxidation [13], wherein the starting radical species is formed through hydrogen atom removal from the phenolic moiety. The phenolic group with an easily abstractable proton and the keto-enol system display significant hydrogen-donating abilities [14] that could initiate the autoxidative degradation of curcumin. The poor metabolic stability of curcumin can be ascribed to the presence of two phenolic hydroxyl groups that are prone to the formation of glucuronide and sulfate conjugates through the phase II metabolic process [15]. Moreover, the enone moiety of curcumin is accessible to nicotinamide adenine dinucleotide phosphate (NADHP) cytochrome P450 reductase or alcohol dehydrogenases, allowing curcumin to be readily reduced to the hydrogenated metabolites dihydrocurcumin, tetrahydrocurcumin, hexahydrocurcumin, and hexahydrocurcuminol [16]. 

Medicinal chemists have performed structural optimizations of curcumin to improve its stability and activity. Most of these attempts were focused on modifying the phenolic hydroxyl groups, keto-enol system, and enone moiety of curcumin [17,18,19,20]. However, the first two abovementioned structural features are required for radical scavenging activity, which is regarded as vital for curcumin chemopreventive and anti-inflammatory activities [21]. In addition, the enone moiety of curcumin, serving as the electrophilic Michael acceptor, can interact with cysteine or selenocysteine residues in proteins to mediate biological activities [22]. Thus, finding a balance between structural stability and biological potency can become challenging. According to the FDA [23], about 30% of the curcumin clinical trials stalled at stage II, mostly due to discrepancies between in vitro activity and in vivo response. Accordingly, to achieve success in clinical trials, obtaining a more definite picture of signaling pathways and ADME profiling for curcumin is recommended. 

**Scheme 2:**
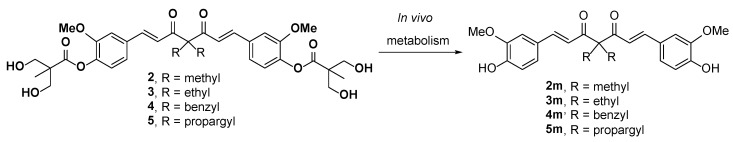
In vivo enzymatic hydrolysis of **2**–**5**.

We previously developed a series of curcuminoid prodrugs by modifying the phenolic hydroxyl groups of curcumin into a 2,2-bis(hydroxymethyl)propionate group [24]. Among the derivatives, the curcumin 2,2-bis(hydroxymethyl)propionate **1** exhibited stability and solubility superior to those of curcumin. Particularly, **1** showed comparable anticancer activity against MDA-MB-231 cells in vitro and in vivo and had 8 to 10 times more potency than curcumin. Subsequently, the α-position in the central 1,3-diketone moiety (C-4 position) of **1** were alkylated with methyl, ethyl, benzyl, and propargyl groups to give compounds **2**, **3**, **4**, and **5**, respectively. Among them, **2**, **3**, and **5** showed more potent in vitro anti-proliferative activity than **1** against HCT-116 cells; therefore, were recommended them as potential therapeutic agents for colon cancer [24]. In our recent in vitro studies on the enzymatic hydrolysis of **1**, we demonstrated that treating **1** with porcine liver esterase (PLE) cleaves its two ester groups gradually to form curcumin (see supporting information). Compounds **2**-**5** are ester-based derivatives and structurally correlated to **1**. Therefore, we predicted that a high concentration of esterase, as particularly found in the intestinal tract and mucosa [25], would hydrolyze **2**, **3**, **4**, and **5** into their metabolites **2m**, **3m**, **4m**, and **5m**, respectively (Scheme 2). However, the anticancer activity of **2m**–**5m** should be evaluated prior to the further in vivo investigations. Compounds **2**–**5** were substituted with two nonpolar alkyl chains at the C-4 position with the loss of keto-enol tautomerism. The anticancer activity of compound **1** derivatives, which were substituted with two hydrophilic units or two polar side chains at the C-4 position, still remains unclear.

Herein, we report the successful replacement of the acidic α-hydrogens at the C-4 position of compound **1** with polar or nonpolar functional groups to produce compounds **6**, **9**, **10**, **11**, **12**, and **14**. Compounds **2m**–**6m** were prepared through the chemical hydrolysis of corresponding parent compounds **2**–**6**. The anticancer activities of **2**–**6**, **9**–**12**, **14** and **2m**–**6m** were evaluated in vitro. A selected compound was submitted for preliminary pharmacokinetic study and antitumor study. The detailed descriptions of the synthetic design, in vitro screening, and in vivo study of the abovementioned compounds are presented as follows.

## 2. Results and Discussion

### 2.1. Chemistry

**Scheme 3:**
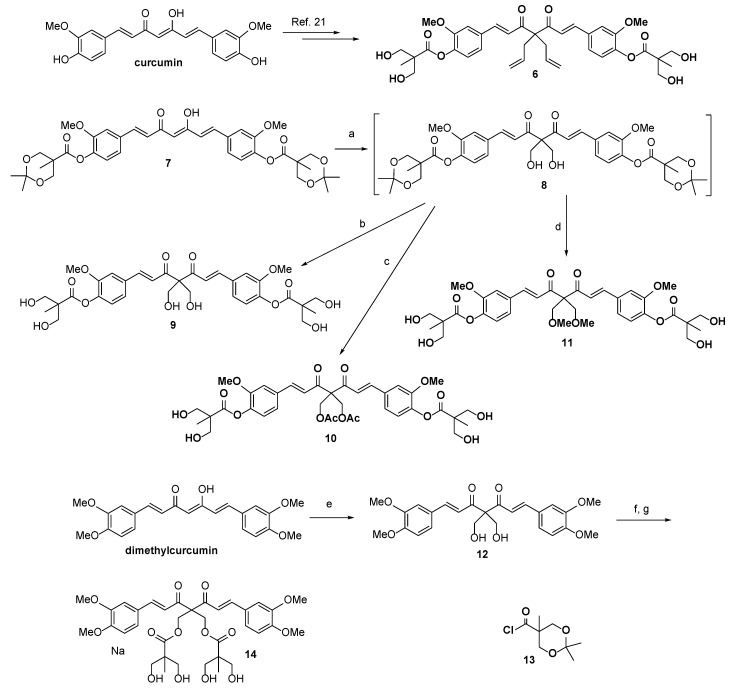
*Reagents and Conditions:* (**a**) Formaldehyde in THF, DBU, 0 °C to rt, 2 h; (**b**) HCl, MeOH, rt, 1 h, 6% for two steps; (**c**) Et_3_N, acetyl chloride, DCM, rt, 12 h; then HCl, MeOH, rt, 1 h, 3% for three steps; (**d**) K_2_CO_3_, CH_3_I, DMF, rt, 6 h; then HCl, MeOH, rt, 1 h, 4% for three steps; (**e**) Formaldehyde in THF, DBU, 0 °C to rt, 2 h, 42%; (**f**) Compound 13, Et_3_N, DCM, rt, 12 h; and (**g**) HCl, MeOH, 1 h, 7% for two steps.

**Scheme 4:**
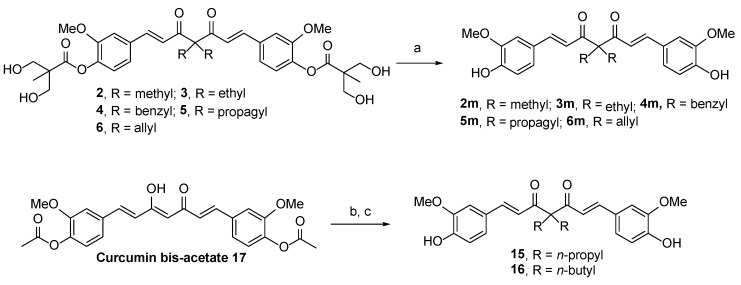
*Reagents and Conditions*: (**a**) NaOMe, MeOH, rt, 2 h, 82–87%; (**b**) K_2_CO_3_, alkyl iodide, DMF, rt, 20 h; and (**c**) NaOMe, MeOH, rt, 3 h, 28–33% for two steps.

The detailed synthesis of compound **1** derivatives (substituted at the C-4 position with allyl (**6**), hydroxymethyl (**10**), acetoxymethyl (**11**), methoxymethyl (**12**)), novel 2,6-dimethyl curcumin (DMC) derivatives (**13** and **15**), and the ester hydrolysis products of derivatives showing potential anticancer activity (**2m**, **3m**, **4m**, **5m**, **6m**, **16**, and **17**) are depicted in Scheme 3 and Scheme 4. As shown in Scheme 3, compound **6** was synthesized according to a procedure established for the synthesis of **2**–**5**. Compound **7**, prepared by the esterification of curcumin with 2,2,5-trimethyl-1,3-dioxane-5-carboxylic acid, was treated with 1,8-diazabicyclo[5.4.0]undec-7-ene (DBU) and formaldehyde in THF to produce intermediary compound **8**. The latter was used without column chromatographic purification in acid-promoted hydrolysis at room temperature to produce **9** with an overall yield of 6% for two synthetic steps.

Compound **9**, however, proved unstable, slowly decomposing even when kept at 4 °C and presenting an NMR spectrum comprising a messy jungle of peaks after being dissolved in DMSO-d_6_ for 1 week at room temperature. The low stability of **9** may have been due to self- or cross-hydrolysis, such that the two hydroxyl groups at the C-4 alkyl chains cleaved the ester linkage of the 2,2-bis(hydroxymethyl)propionate group. Previously, we had found that upon exposure to alcohol for several hours, the ester linkages of **1** were broken under neutral or basic conditions, leading to the conversion of **1** to curcumin and several unknown products. Therefore, we modified the hydroxymethyl group of **9** and were able to produce compounds **10** and **11**. To prepare **10**, compound **9** was acetylated with acetyl chloride in the presence of Et_3_N, and the resulting di-acetylation intermediate was hydrolyzed by hydrochloric acid. Compound **11** was obtained with an overall yield of 5% following a similar three-step reaction modified for methylation. The stability of these two products at room temperature may have been due to the conversion of the labile hydroxyl groups of the C-4 alkyl chains of **10** and **11** into non-nucleophilic methyl and acetyl groups, respectively, which prevented degradation.

Because DMC is a dimethylated derivative of curcumin [26] that exhibits better anticancer activity and stability than curcumin, we synthesized a novel DMC derivative (compound **12**) in which C-4 was substituted with two bulky 2,2-bis(hydroxymethyl)propionate groups. To produce this derivative, commercially available DMC was reacted with DBU and formaldehyde in THF. The reaction of **12** with 2,2,5-trimethyl-1,3-dioxane-5-carbonyl chloride **13** [27] in the presence of Et_3_N provided a diol intermediate, which was then hydrolyzed by hydrochloric acid in MeOH to yield compound **14**. In addition, as illustrated in Scheme 4, compounds **2m**–**6m** were prepared through the NaOMe-mediated hydrolysis of their corresponding parent compounds **2**–**6**, with high yield.

**Scheme 5:**
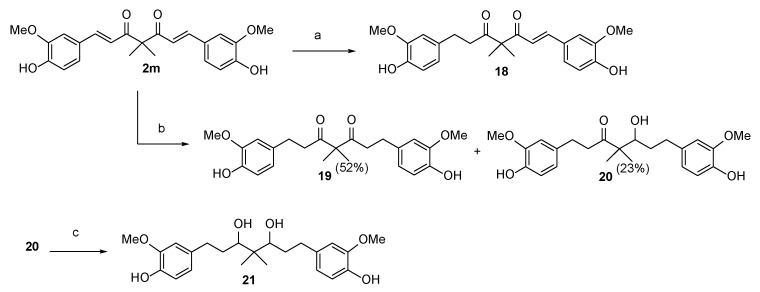
*Reagents and Conditions*: (**a**) 10% *w*/*w* Pd/C, ethyl acetate, rt, 1 h, 19%; (**b**) 10% *w*/*w* Pd/C, MeOH, rt, 2 h; and (**c**) NaBH_4_, MeOH, 0 °C to rt, 2 h, 31%.

For SAR analysis, compounds **15** and **16**, which were substituted with two straight alkyl chains with three or four carbons at the C4 position, were prepared from curcumin bisacetate **17** [28] via a two-step procedure comprising K_2_CO_3_-induced dialkylation and NaOMe-mediated hydrolysis. For the synthesis of four possible phase I metabolites of compound **2**, compound **2m** was subjected to hydrogenation reactions. As shown in Scheme 5, compound **18** is a partially hydrogenated product that can be prepared in 19% yield by treating compound **2m** with H_2(g)_ (1.0 atm, balloon) and a catalytic amount of 10% *w*/*w* Pd/C in ethyl acetate. When the solvent was changed from ethyl acetate to methanol, the hydrogenation reaction yielded the fully hydrogenated diketone **19** in 52% yield and β-hydroxy ketone **20** in 23% yield. Finally, the reduction of **20** with NaBH_4_ in MeOH at room temperature (rt) for 2 h provided the desired diol compound **21** in 31% yield.

### 2.2. Structure–Activity Relationship

As mentioned above, we synthesized **2**–**5** and evaluated their anti-proliferative activity against the MDA-MB-231 and HCT-116 cell lines (72-h treatment). These compounds, along with all newly synthesized compounds, were screened for anti-proliferative activity against the same TNBC and colon cancer cell lines. The results of the in vitro assay are compiled in Table 1.

The anticancer effects of **2**–**5** against both cell lines were still better or at least similar to that of **1**, although the drug treatment duration was reduced to 48 h. The newly synthesized compound **6** had a higher anti-proliferative activity against HCT-116. Compound **9** was less potent against the cell lines than curcumin and **1**, which was used as a reference compound in the current study. We hypothesize that the two polar and hydrophilic side chains at the C-4 position in **9** are the cause for the weak anticancer effects observed for this compound. Another possibility is that **9** decomposed during the screening due to its low stability. Compound **10**, having two acetoxymethyl groups rather than hydroxymethyl groups at the C-4 side chains, showed significant improvement in in vitro anticancer activities compared to **9**; however, it was still less potent than **1**. Compound **11**, substituted with two methoxymethyl groups at the C-4 position, displayed poor anticancer activity with high IC_50_ values for both cell lines used.

After listing the compounds in descending order based on their potency against HCT-116 (**6** > **3** > **2** > **5** > **1** > **10** > **4** >> **11** >> **9**), we concluded that higher polarity and hydrophilicity of the C-4 side chain are detrimental to the anticancer activity of compound **1** derivatives. Alternatively, the DMC derivative 4,4-dihydroxymethyl dimethoxycurcumin (compound **12**) displayed low anti-proliferative activity. When the two hydroxyl moieties of **12** both esterified with 2,2-bis(hydroxymethyl)propionic acid, the resulting compound **14** exhibited activities comparable to those of **12**, which correlates with our previous findings regarding the unfavorable effect of having a hydrophilic moiety at the C-4 position.

Subsequently, the activities of the ester hydrolysis products **2m**–**6m** were explored. Unexpectedly, **5m** displayed substantial improvement in anticancer activity by two- to four-fold relative to that of parent **5**. Compound **2m** exhibited activity similar to that of **2**, whereas **3m**, **4m**, and **6m** showed inferior activity than their corresponding parent compounds **3**, **4**, and **6**. Further screening of **15** and **16** revealed an obvious SAR, such that the anticancer activity of these hydrolysis compounds decreased with the increasing length of the C-4 alkyl side chains. In addition, lower structural volume was seemingly preferred to sterically congested side chains, as demonstrated by the analysis of **2m**, **3m**, **15**, **4m**, and **16**.

**Scheme 6:**
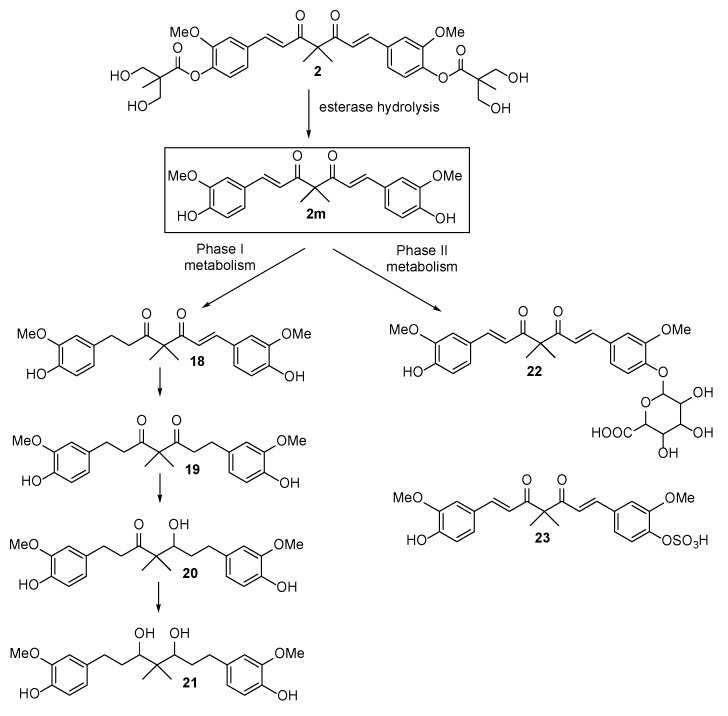
In vivo metabolism of **2**.

Among all examined compounds, **2** and its ester hydrolysis product **2m** possessed significant anticancer activity against MDA-MB-231 and HCT-116; therefore, **2** was chosen for further studies. Notably, the solubility of **2** was more significant than that of curcumin as was evident from the vehicle of **2** for the following in vivo study. The maximum solubility of **2** in the aqueous vehicle comprising 5% ethanol, 10% Tween 80, and 85% saline was approximately 200 mg/mL, while curcumin was almost insoluble in the vehicle. Based on the logic for the metabolism of curcumin [29] and an ester-type prodrug [30], we speculated that the in vivo metabolism of **2** would readily produce **2m**. Subsequently, the latter would be converted into glucuronide **22** and sulfate **23** through the phase II metabolism conjugation of hydrogenated products **18**, **19**, **20**, and **21** via the phase I metabolic pathway (Scheme 6). To verify our assumptions, the preliminary pharmacokinetic evaluation of **2** was executed.

### 2.3. The Preliminary Pharmacokinetic Evaluation of 2

The preliminary in vivo pharmacokinetic evaluation of **2** was designed to identify the anticipated metabolites and evaluate the plasma stability of **2** and **2m**. The four possible phase I metabolites **18**, **19**, **20**, and **21** were synthesized and subjected to anti-proliferative screening against MDA-MB-231 and HCT-116. Compound **18** exhibited moderate to weak anti-proliferative activity (MDA-MB-231, IC_50_ = 36.48 ± 0.34 μM; HCT-116, IC_50_ = 9.64 ± 0.21 μM) and the others are inactive against both cell lines (IC_50_ ≥ 100 μM). The assessment of **2** and relevant metabolites was performed in male Sprague-Dawley *rats* after the oral administration of **2** at a dose of 100 mg/kg. The LC signals of **2**, **2m**, and **18**–**21** was recognized unequivocally by comparison to the signals of standard compounds. The direct analysis of **22** and **23** was not easily attainable due to the low extraction efficiency and high probability of sample loss in LC column. It has been well-documented that curcumin glucuronide and curcumin sulfate can be transformed back into curcumin by the enzyme-mediated hydrolysis reaction [31]. Accordingly, the treatment of **22** and **23** with enzyme that contains sulfatase and glucuronidase is supposed to produce **2m**. In the current study, the amounts of **22** and **23** were not calculated individually but counted as a summary value based on the disparities between the LC signal of **2m** in enzyme-treated and -untreated serum samples.

**Figure 1:**
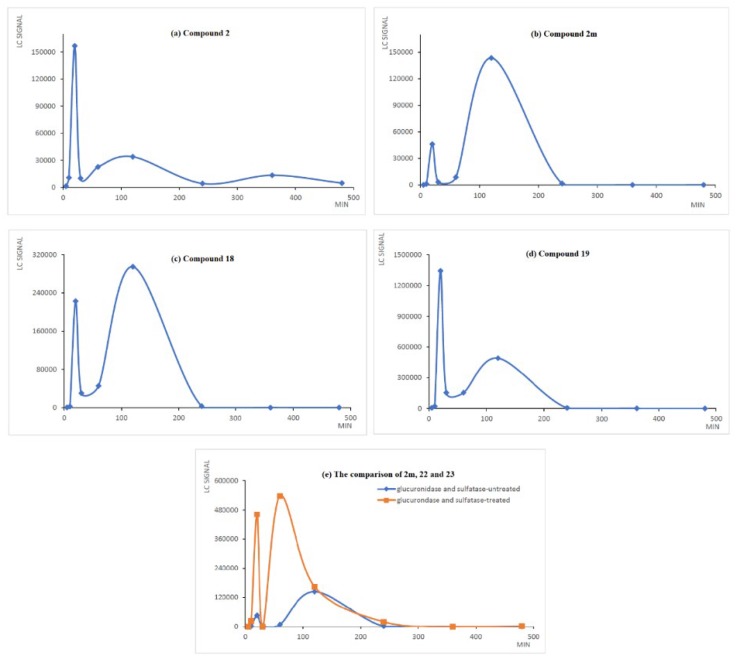
The amount of **2**, **2m**, **18**, **19**, **22**, and **23** in serum at different time points following a single oral administration of **2** at a dose of 100 mg/kg. The blood samples were collected at 5, 10, 20, 30, 60, 120, 240, 360, and 480 min after the administration of **2**. The samples were deproteinated and analyzed by HPLC.

In practice, LC-MS analysis of one serum sample which was collected at 20 min post-dosing demonstrated the existence of **2**, **2m**, **18**, **19**, **20**, **21**, **22** and **23** (Figure S1 in the Supporting Information). Shown in Figure 1 are the LC signal count-time profiles for **2**, **2m**, **18**, **19**, and the comparison of **2m**, **22**, and **23**, following oral administration. The analysis of **20** and **21** is not illustrated due to their low LC signal response and non-significant anticancer activity. The maximum blood content of **2** was achieved at 20 min, while the second and third highest appeared at 120 min and 360 min, respectively, following administration. The multiple peaks of **2** observed in the profile could be attributed to an enterohepatic circulation cycle. 

Such behavior, extensively studied in the phenolic drugs, was beneficial in maintaining the circulating concentration of free-form curcumin [32] and may extend the pharmacological effect of drug [33]. The hydrolysis metabolite **2m** emerged from 10 min and reached its maximum blood content at 120 min, indicating that a medium-to-high esterase-mediated biotransformation proceeded in vivo to yield the efficient absorption of **2m**. Subsequently, the phase II metabolic process of **2m** progressed quickly to produce **22** and **23**. The ratio of **2m** to its phase II metabolites (**22** and **23**) is about 1:4 based on the area under the curves in part (e) of Figure 1.

The LC signal count-time profiles of **18** and **19** are analogous to that of **2m**, implying that phase I reduction reaction also occurred rapidly. Compared to non-formulated curcumin with a short elimination period of 28 min in the rat model [34], **2** exhibited superior plasma stability. Otherwise, the active metabolite **2m** is equally potent as **2** with regard to anticancer activity, and the maximum blood content of **2m** appeared after that of **2** could be considered as an extension of drug efficacy of **2**. Thus, the pharmacokinetic outcome was to guide the following in vivo efficacy study of **2**.

### 2.4. In Vivo Antitumor Efficacy of 2

**Figure 2:**
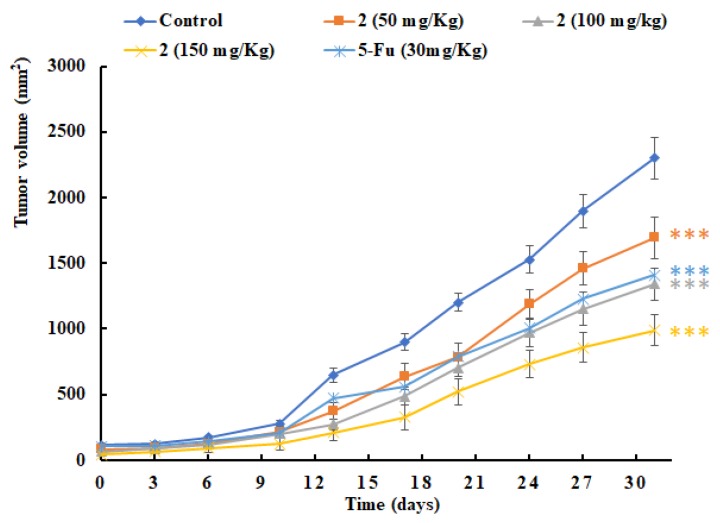
The efficacy study of **2**. *In vivo* antitumor activity of compound **2** was evaluated in HCT-116 tumor xenograft bearing nude mice. Compound **2** (50, 100, or 150 mg/kg, p.o.) was administered once daily and 5-Fluorouracil was administrated (30 mg/kg, i.p.) every other day. The results are the mean ± SEM of six mice in each group. *** *P* < 0.001 compared to untreated control.

**Figure 3:**
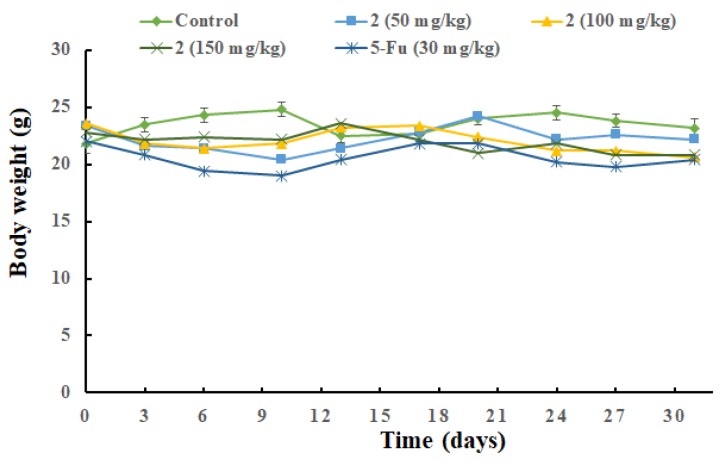
Mean body weight-time profile of **2**- administrated and control groups.

The therapeutic effect of **2** was evaluated on HCT-116 colon tumor xenograft bearing nude mice. The oral administration of **2** at doses of 50, 100, or 150 mg/kg bw per day were performed after tumors reached approximately 100 mm^3^ and continued for 30 consecutive days. 5-Fluorouracil treatment (30 mg/kg bw, i.p., QOD) was used as a positive control. The group treated with compound **2** (100 mg/kg) had a statistically significant reduction in tumor volumes, with a 45% tumor inhibition ratio observed when compared with the cancerous control mice. Positive control group exhibited a capability to cause 40% tumor-growth inhibition. There was a clear dose- and time-dependent inhibitory effect on tumor size in the other two groups, in which at 50 and 150 mg/kg doses the volume of the HCT-116 colon tumor was reduced to 73% and 40% of the control, respectively (Figure 2). The body weight of the mice was recorded prior to dosing every 3 d, and the average body weight of each group is depicted in Figure 3. Otherwise, they were monitored for visible signs of toxicity and behavioral changes 1 h after each administration. No obvious adverse effects were observed between the **2**-administrated and control groups.

## 3. Materials and Methods

### 3.1. General Information

The reactions were performed under an air atmosphere unless otherwise stated. All solvents and reagents were employed as received. Analytical thin layer chromatography (TLC) was performed on SiO_2_ 60 F_254_ plates and flash column chromatography was carried out using SiO_2_ 60 (particle size 0.040–0.055 mm, 230–400 mesh), both of which are available from E. Merck (Darmstadt, Germany). Visualization was performed under UV irradiation at 254 nm followed by staining with aqueous potassium permanganate [KMnO_4_ (3 g) and K_2_CO_3_ (20 g) in 300 mL of H_2_O containing 5 mL of an aqueous solution of NaOH (5%, *w*/*v*)] and charring by heat gun. ^1^H- and ^13^C-NMR spectra were recorded on a 500 FT NMR instrument (Bruker, Billerica, MA, USA). Chloroform-*d* and methanol-*d* were used as solvents and TMS (*δ* = 0.00 ppm) as an internal standard. Chemical shifts are reported as *δ* values in ppm as referenced to TMS. Multiplicities are recorded as s (singlet), d (doublet), t (triplet), q (quartet), quint (quintet), sext (sextet), sept (septet), dd (doublet of doublets), dt (doublet of triplets), br (broad), m (multiplet). Coupling constants (*J*) are expressed in Hz. LRMS and HRMS were measured by a JMS-HX110 spectrometer (JEOL, Tokyo, Japan) and spectroscopic data were recorded as *m*/*z* values. Melting points were measured using an Electrothermal instrument (Anatec Yanaco Inc., Kyoto, Japan).

#### 3.1.1. *[(1E,6E)-4,4-Diallyl-3,5-dioxohepta-1,6-diene-1,7-diyl]bis(2-methoxy-4,1-phenylene) bis[3-hydroxy-2-(hydroxymethyl)-2-methylpropanoate]* (**6**)

Mp = 195–197 °C; ^1^H-NMR (DMSO-d_6_) δ 7.59 (d, *J* = 15.5 Hz, 2H), 7.48 (s, 2H), 7.35 (dd, *J* = 8.2, 1.5 Hz, 2H), 7.12–7.04 (m, 4H), 5.59–5.52 (m, 2H), 5.12–5.04 (m, 4H), 4.82–4.80 (m, 4H), 3.78 (s, 6H), 3.60 (s, 8H), 2.84 (d, *J* = 7.2 Hz, 4H), 1.17 (s, 6H); ^13^C NMR (DMSO-d_6_) δ 196.8, 173.3, 151.7, 143.1, 142.1, 133.3, 133.2, 123,9, 122.9, 122.8, 119.5, 113.4, 67.4, 64.0, 56.7, 51.0, 35.0, 17.3; HRMS [ESI]^+^ calculated for C_37_H_45_O_12_: 681.2911 [M + H]^+^; found: 681.2903.

#### 3.1.2. *[(1E,6E)-4,4-bis(Hydroxymethyl)-3,5-dioxohepta-1,6-diene-1,7-diyl]bis(2-methoxy-4,1-phenylene) bis(2,2,5-trimethyl-1,3-dioxane-5-carboxylate)* (**8**)

To a stirred solution of **7** (1.00 g, 1.47 mmol) in THF (7.3 mL, containing 0.6 M formaldehyde) was added DBU (38.9 mg, 0.04 mmol) at 0 °C. The reaction mixture was stirred at the same temperature for 30 min and then warm to room temperature. After 2 h, the solution was concentrated under vacuum to provide crude compound, which was identified by LRMS [ESI]^+^ calculated for C_39_H_49_O_14_: 741.31 [M + H]^+^; found: 741.33 and used for next step without purification.

#### 3.1.3. *[(1E,6E)-4,4-bis(Hydroxymethyl)-3,5-dioxohepta-1,6-diene-1,7-diyl]bis(2-methoxy-4,1-phenylene) bis[3-hydroxy-2-(hydroxymethyl)-2-methylpropanoate]* (**9**)

A solution of crude **8** (0.34 g) and 6 N HCl (0.15 mL) in MeOH (1.83 mL) was stirred at room temperature for 1 h. The reaction mixture was concentrated under vacuum to give the crude product, which was purified by flash chromatography on silica gel with MeOH/CH_2_Cl_2_ (1:20) to afford compound **9** (40 mg) as an orange oil. Yield 6% for two steps. ^1^H-NMR (DMSO-d_6_) δ 7.52 (d, *J* = 15.6 Hz, 2H), 7.44 (s, 2H), 7.30 (d, *J* = 8.2 Hz, 2H), 7.08–7.04 (m, 4H), 5.16 (br s, 6H), 4.19 (s, 4H), 3.78 (s, 6H), 3.61 (s, 8H), 1.17 (s, 6H); LRMS [ESI]^+^ calculated for C_33_H_40_O_14_: 661.25 [M + H]^+^; found: 661.4.

#### 3.1.4. *[(1E,6E)-4,4-bis(Acetoxymethyl)-3,5-dioxohepta-1,6-diene-1,7-diyl]bis(2-methoxy-4,1-phenylene) bis[3-hydroxy-2-(hydroxymethyl)-2-methylpropanoate]* (**10**)

To a stirred solution of crude **8** (0.450 g) in DCM (10 mL) were added NEt_3_ (1.0 mL) and acetyl chloride (0.140 g, 1.81 mmol) at 0 °C. The reaction mixture was warmed to room temperature and stirred for 12 h. Then H_2_O (10 mL) and diluted HCl_(aq)_ (1 *N*, 3 mL) were added to quench the reaction. The aqueous layer was separated and extracted with CH_2_Cl_2_ (3 × 8 mL). The combined organic extracts were washed with brine, dried over MgSO_4_, filtered and concentrated to give the crude product, which was then treated with HCl_(aq)_ (6 *N*, 0.2 mL) in MeOH (2 mL) at room temperature. The reaction mixture was stirred at room temperature for 1h and then concentrated under vacuum to give the crude product, which was purified on silica gel with MeOH/CH_2_Cl_2_ (1:20) to afford **10** (33 mg) as a yellow solid (mp = 194–196 °C). Yield 3% for three steps; ^1^H-NMR (DMSO-d_6_) δ 7.68 (d, *J* = 15.5 Hz, 2H), 7.51 (s, 2 H), 7.37 (d, *J* = 8.0 Hz, 2H), 7.20 (d, *J* = 15.5 Hz, 2H), 7.08 (d, *J* = 8.5 Hz, 2H), 4.85 (t, *J* = 5.0 Hz, 8H), 3.80 (s, 6 H), 3.65-3.59 (m, 8H), 1.97 (s, 6H), 1.19 (s, 6H); ^13^C-NMR (DMSO-d_6_) δ 193.1, 173.3, 170.5, 151.7, 144.6, 142.5, 132.9, 123.9, 122,7, 121.7, 113.6, 64.0, 61.9, 56.7, 51.0, 20.9, 17.3; HRMS [ESI]^+^ calculated for C_37_H_44_NaO_16_: 767.2527 [M + Na]^+^; found: 767.2521.

#### 3.1.5. *[(1E,6E)-4,4-bis(Methoxymethyl)-3,5-dioxohepta-1,6-diene-1,7-diyl]bis(2-methoxy-4,1-phenylene) bis[3-hydroxy-2-(hydroxymethyl)-2-methylpropanoate]* (**11**)

To a stirred solution of crude **8** (0.453 g) in DMF (1.0 mL) were added K_2_CO_3_ (0.240 g, 1.73 mmol) and MeI (0.247 g, 1.73 mmol) at 0 ^o^C. The reaction mixture was warmed to room temperature and stirred for 6 h. Then H_2_O (10 mL) and diluted HCl_(aq)_ (1 *N*, 3 mL) were added to quench the reaction. The aqueous layer was separated and extracted with EtOAc (3 × 8 mL). The combined organic extracts were washed with brine, dried over MgSO_4_, filtered and concentrated to give the crude product, which was then treated with HCl_(aq)_ (6 *N*, 0.25 mL) in MeOH (2 mL) at room temperature. The reaction mixture was stirred at room temperature for 1h and then concentrated under vacuum to give the crude product, which was purified on silica gel with MeOH/CH_2_Cl_2_ (1:20) to afford **11** (73 mg) as a yellow solid (mp = 187–188 °C). Yield 5% for three steps; ^1^H-NMR (DMSO-d_6_) δ 7.65 (d, *J* = 16.0 Hz, 2H), 7.49 (s, 2 H), 7.31 (d, *J* = 10.0 Hz, 2H), 7.08 (d, *J* = 8.1 Hz, 2H), 6.69 (d, *J* = 16.0 Hz, 2H), 4.85 (t, *J* = 5.5 Hz, 4 H), 3.80 (s, 6H), 3.74 (s, 6 H), 3.66-3.60 (m, 8H), 1.20 (s, 6H); ^13^C-NMR (DMSO-d_6_) δ 193.1, 173.4, 167.1, 151.6, 144.4, 141.8, 133.2, 123.8, 121,9, 118.4, 112.6, 64.0, 56.3, 51.9, 51.0, 17.3; HRMS [ESI]^+^ calculated for C_35_H_44_NaO_14_: 711.2629 [M + Na]^+^; found: 711.2633.

#### 3.1.6. *(1E,6E)-1,7-bis(3,4-Dimethoxyphenyl)-4,4-bis(hydroxymethyl)hepta-1,6-diene-3,5-dione* (**12**)

To a stirred solution of DMC (1.270 g, 3.20 mmol) in THF (20 mL, containing 0.6 M formaldehyde) was added DBU (15 mg, 0.10 mmol). The reaction mixture was stirred at 0 °C for 30 min. The reaction mixture was concentrated under vacuum and then purified by flash chromatography on silica gel with MeOH/CH_2_Cl_2_ (1:32) to afford **12** (558 mg, 42% yield) as a yellow solid; mp = 174–176 °C; ^1^H-NMR (DMSO-d_6_) δ 7.49 (d, *J* = 15.5 Hz, 2H), 7.48 (d, *J* = 2.0 Hz, 2H), 7.24 (d, *J* = 8.5 Hz, 2H), 6.97–6.91 (m, 4H), 4.78 (t, *J* = 4.5 Hz, 2H), 4.19 (d, *J* = 4.5 Hz, 4H), 3.79 (s, 12H); ^13^C-NMR (DMSO-d_6_) δ 196.2, 151.7, 149.1, 142.8, 127.4, 123.8, 120,9, 112.1, 111.3, 71.6, 60.1, 56.1, 56.0; HRMS [ESI]^+^ calculated for C_25_H_28_NaO_8_: 479.1682 [M + Na]^+^; found: 479.1677.

#### 3.1.7. *2,2-bis[(E)-3-(3,4-Dimethoxyphenyl)acryloyl]propane-1,3-diylbis[3-hydroxy-2-(hydroxymethyl)-2-methylpropanoate]* (**14**)

To a solution of **12** (0.170 g, 0.37 mmol) in CH_2_Cl_2_ (4 mL) was added NEt_3_ (0.15 mL, 1.10 mmol), DMAP (0.110 g, 0.92 mmol) and 2,2,5-trimethyl-1,3-dioxane-5-carbonyl chloride **13** (0.210 g, 1.10 mmol) sequentially at room temperature. The reaction mixture was stirred at the same temperature for 12h and then concentrated under vacuum to give the crude product, which without purification was treated with HCl_(aq)_ (6 *N*, 0.1 mL) in MeOH (0.5 mL). The resulting mixture was stirred at room temperature for 1h and then concentrated under vacuum to give the crude product, which was purified by flash chromatography on silica gel with MeOH/CH_2_Cl_2_ (1:19) to afford compound **14** (16 mg, 7% for two steps) as a yellow solid; mp > 300 °C; ^1^H-NMR (DMSO-d_6_) δ 7.64 (d, *J* = 15.5 Hz, 2H), 7.34–7.30 (m, 4H), 7.03 (d, *J* = 15.0 Hz, 2H), 6.99 (d, *J* = 8.0 Hz, 2H), 4.74 (s, 4H), 4.63 (t, *J* = 5.5 Hz, 4H), 3.79 (s, 12H), 3.40-3.37 (m, 8H), 0.95(s, 6H); ^13^C-NMR (DMSO-d_6_) δ 193.3, 174.6, 152.1, 149.4, 145.0, 127.1, 124.7, 119,5, 112.0, 111.4, 66.9, 63.9, 62.3, 56.1, 56.1, 50.7, 17.0; HRMS [ESI]^+^ calculated for C_35_H_44_NaO_14_: 711.2629 [M + Na]^+^; found: 711.2620.

#### 3.1.8. General Procedure for the Synthesis of Compounds **2m**–**6m**

The general procedure is illustrated immediately below with compound **2m** as a specific example.

##### *(1E,6E)-1,7-bis(4-Hydroxy-3-methoxyphenyl)-4,4-dimethylhepta-1,6-diene-3,5-dione* (**2m**)

To a stirred solution of compound **2** (0.450 g, 0.72 mmol) in CH_3_OH (6 mL) were added NaOMe (0.116 g, 2.16 mmol) at room temperature. The reaction mixture was stirred at the same temperature for 2 h and then H_2_O (5 mL) and diluted HCl_(aq)_ (1 *N*, 1 mL) were added to quench the reaction. The aqueous layer was separated and extracted with CH_2_Cl_2_ (3 × 6 mL). The combined organic extracts were washed with brine, dried over MgSO_4_, filtered and concentrated to give the crude product, which was then purified by flash chromatography on silica gel with EtOAc/*n*-hexane/CH_2_Cl_2_ (1:2:1) to afford **2m** (0.245 g, 86% yield) as an orange solid; mp = 104−106 °C; ^1^H-NMR (CDCl_3_) δ 7.69 (d, *J* = 15.5 Hz, 2H), 7.12 (dd, *J* = 8.0, 1.5 Hz, 2H), 7.01 (s, 2H), 6.91 (d, *J* = 8.5 Hz, 2H), 6.65 (d, *J* = 15.5 Hz, 2H), 5.97 (s, 2H), 3.94 (s, 6H), 1.48 (s, 6H); ^13^C-NMR (CDCl_3_) δ 198.2, 148.6, 146.8, 144.5, 126.8, 124.1, 119.1, 114.8, 109.8, 60.8, 56.0, 14.1; HRMS [ESI]^+^ calculated for C_23_H_24_NaO_6_: 419.1471 [M + Na]^+^; found: 419.1480

##### *(1E,6E)-4,4-Diethyl-1,7-bis(4-hydroxy-3-methoxyphenyl)hepta-1,6-diene-3,5-dione* (**3m**)

Yield: 87%; mp = 114–115 °C; ^1^H-NMR (CDCl_3_) δ 7.64 (d, *J* = 15.5 Hz, 2H), 7.05 (dd, *J* = 8.3, 1.5 Hz, 2H), 6.95 (d, *J* = 2.0 Hz, 2H), 6.85 (d, *J* = 8.0 Hz, 2H), 6.59 (d, *J* = 15.5 Hz, 2H), 5.88 (s, 2H), 3.88 (s, 6H), 2.06 (q, *J* = 7.5 Hz, 4H), 0.71 (t, *J* = 7.5 Hz, 6H); ^13^C-NMR (CDCl_3_) δ 197.9, 148.5, 146.7, 144.0, 126.8, 124.2, 119.6, 114.7, 109.7, 69.0, 56.1, 21.6, 7.7; HRMS [ESI]^+^ calculated for C_25_H_28_NaO_6_: 447.1784 [M + Na]^+^; found: 447.1788.

##### *(1E,6E)-4,4-Dibenzyl-1,7-bis(4-hydroxy-3-methoxyphenyl)hepta-1,6-diene-3,5-dione* (**4m**)

Yield: 82%; mp = 134–135 °C; ^1^H-NMR (CDCl_3_) δ 7.72 (d, *J* = 15.0 Hz, 2H), 7.26–7.21 (m, 6H), 7.15–7.13 (m, 4H), 7.07–7.05 (m, 2H), 6.90–6.89 (m, 4H), 6.58 (dd, *J* = 15.5, 1.0 Hz, 2H), 5.31 (s, 2H), 3.89 (s, 6H), 3.42 (s, 4H); ^13^C-NMR (CDCl_3_) δ 196.9, 148.7, 146.8, 144.0, 136.6, 130.4, 128.1, 126.8, 126.6, 124.3, 120.8, 114.8, 109.8, 70.1, 56.1, 37.7; HRMS [ESI]^+^ calculated for C_25_H_32_NaO_6_: 571.2097 [M + Na]^+^; found: 571.2090.

##### *(1E,6E)-1,7-bis(4-Hydroxy-3-methoxyphenyl)-4,4-di(prop-2-yn-1-yl)hepta-1,6-diene-3,5-dione* (**5m**)

Yield: 83%; mp = 201–203 °C; ^1^H-NMR (CDCl_3_) δ 7.75 (d, *J* = 15.0 Hz, 2H), 7.13 (dd, *J* = 8.0, 1.5 Hz, 2H), 7.03 (d, *J* = 2.0 Hz, 2H), 6.92 (d, *J* = 8.0 Hz, 2H), 6.67 (d, *J* = 15.0 Hz, 2H), 5.97 (s, 2H), 3.95 (s, 6H), 3.19 (s, 4H), 2.04 (s, 4H); ^13^C-NMR (CDCl_3_) δ 193.5, 148.9, 145.9, 126.5, 124.7, 117.8, 114.8, 109.8, 79.1, 71.9, 66.9, 56.1, 21.2; HRMS [ESI]^+^ calculated for C_27_H_28_O_6_: 471.1784 [M + Na]^+^; found: 471.1783.

##### *(1E,6E)-4,4-Diallyl-1,7-bis(4-hydroxy-3-methoxyphenyl)hepta-1,6-diene-3,5-dione* (**6m**)

Yield: 85%; mp = 160–162 °C; ^1^H-NMR (CDCl_3_) δ 7.65 (d, *J* = 15.5 Hz, 2H), 7.06 (dd, *J* = 8.3, 1.5 Hz, 2H), 6.95 (d, *J* = 2.0 Hz, 2H), 6.85 (d, *J* = 8.0 Hz, 2H), 6.61 (d, *J* = 15.5 Hz, 2H), 5.87 (s, 2H), 5.54–5.49 (m, 2H), 5.07–5.04 (m 4H), 3.88 (s, 6H), 2.78 (d, *J* = 7.5 Hz, 4H); ^13^C-NMR (CDCl_3_) δ 194.6, 148.7, 146.8, 144.6, 132.3, 126.7, 119.3, 119.1, 114.7, 109.7, 67.8, 56.1, 34.4; HRMS [ESI]^+^ calculated for C_27_H_24_NaO_6_: 467.1471 [M + Na]^+^; found: 467.1477.

#### 3.1.9. General Procedure for the Synthesis of Compounds **15** and **16**

The general procedure is illustrated below with compound **15** as a specific example. To a solution of curcumin bisacetate **17** (0.76 g, 1.68 mmol) in DMF (8 mL) was added K_2_CO_3_ (0.58 g, 4.20 mmol) and propyl bromide (0.435 g, 3.53 mmol) sequentially at 0 °C. The reaction mixture was stirred at room temeperatre for 20 h and then H_2_O (10 mL) and diluted HCl_(aq)_ (1 *N*, 5 mL) were added to quench the reaction. The aqueous layer was separated and extracted with EtOAc (3 × 10 mL). The combined organic layer was dried over MgSO_4_, filtered and concentrated to give the crude product, which was subjected without any purification to the base-promoted hydrolysis reaction [NaOMe (0.190 g, 3.53 mmol), MeOH (5 mL)]. The reaction was followed by TLC until no starting material was present. The resulting mixture was then concentrated to give the crude product, which was then purified by flash chromatography on silica gel with EtOAc/*n*-hexane/CH_2_Cl_2_ (1:2:1) to afford compound **15** (0.213 g, 28% yield for two steps) as a yellow solid.

##### *(1E,6E)-1,7-bis(4-Hydroxy-3-methoxyphenyl)-4,4-dipropylhepta-1,6-diene-3,5-dione* (**15**)

mp = 137–139 °C; ^1^H-NMR (CDCl_3_) δ 7.69 (d, *J* = 15.0 Hz, 2H), 7.11 (dd, *J* = 8.3, 1.5 Hz, 2H), 7.01 (d, *J* = 1.5 Hz, 2H), 6.91 (d, *J* = 8.0 Hz, 2H), 6.65 (d, *J* = 15.5 Hz, 2H), 5.90 (s, 2H), 3.94 (s, 6H), 2.05–2.01 (m, 4H), 1.15–1.08 (m, 4H), 0.95 (t, *J* = 7.5 Hz, 6H); ^13^C-NMR (CDCl_3_) δ 197.9, 148.5, 146.7, 144.0, 126.8, 124.1, 119.5, 114.7, 109.7, 68.4, 56.1, 31.8, 16.8, 14.7; HRMS [ESI]^+^ calculated for C_27_H_32_NaO_6_: 475.2097 [M + Na]^+^; found: 475.2100.

##### *(1E,6E)-4,4-Dibutyl-1,7-bis(4-hydroxy-3-methoxyphenyl)hepta-1,6-diene-3,5-dione* (**16**)

mp = 144–146 °C; ^1^H-NMR (CDCl_3_) δ 7.68 (d, *J* = 15.5 Hz, 2H), 7.11 (dd, *J* = 8.3, 1.5 Hz, 2H), 7.01 (s, 2H), 6.91 (d, *J* = 8.2 Hz, 2H), 6.65 (d, *J* = 15.5 Hz, 2H), 6.01 (s, 2H), 3.93 (s, 6H), 2.07–2.03 (m, 4H), 1.37–1.33 (m, 4H), 1.10–1.03 (m, 4H), 0.90 (t, *J* = 7.3 Hz, 6H); ^13^C-NMR (CDCl_3_) δ 198.0, 148.5, 146.8, 144.0, 126.9, 124.1, 119.5, 114.7, 109.7, 68.3, 56.1, 29.1, 25.5, 23.2, 13.9; HRMS [ESI]^+^ calculated for C_29_H_36_NaO_6_: 503.2410 [M + Na]^+^; found: 503.2400.

#### 3.1.10. *(E)-1,7-bis(4-Hydroxy-3-methoxyphenyl)-4,4-dimethylhept-1-ene-3,5-dione* (**18**)

To a stirred solution of compound **2m** (0.740 g, 1.87 mmol) in EtOAc (10 mL) was added Pd/C (37 mg, 5% *w*/*w*) in one portion. The resulting mixture was hydrogenated under 1 atmosphere of H_2_ at room temperature. After 1 h, the mixture was filtered by celite and concentrated to give the crude product, which was purified by flash chromatography on silica gel with EtOAc/*n*-hexane/CH_2_Cl_2_ (1:2:1) to afford compound **18** (140 mg, 19% yield) as a light-yellow oil and recover compound **2m** (500 mg, 67% yield). ^1^H-NMR (CDCl_3_) δ 7.61 (d, *J* = 15.5 Hz, 1H), 7.07 (dd, *J* = 8.5, 1.5 Hz, 1H), 6.95–6.93 (m, 2H), 6.75 (d, *J* = 8.0 Hz, 1H), 6.65–6.62 (m, 2H), 6.49 (d, *J* = 15.5 Hz, 1H), 6.07 (s, 1H), 5.45 (s, 1H), 3.94 (s, 3H), 3.79 (s, 3H), 2.83 (t, *J* = 7.5 Hz, 2H), 2.74 (t, *J* = 7.0 Hz, 2H), 1.38 (s, 6H); ^13^C-NMR (CDCl_3_) δ 209.4, 197.7, 148.7, 146.8, 146.3, 144.7, 143.8, 132.6, 126.9, 123.9, 121.0, 118.2, 114.9, 114.2, 111.0, 110.0, 61.7, 56.0, 55.7, 40.7, 29.6, 21.0; HRMS [ESI]^+^ calculated for C_23_H_26_NaO_6_: 421.1627 [M + Na]^+^; found: 421.1233.

#### 3.1.11. *1,7-bis(4-Hydroxy-3-methoxyphenyl)-4,4-dimethylheptane-3,5-dione* (**19**) *and 5-hydroxy-1,7-bis(4-hydroxy-3-methoxyphenyl)-4,4-dimethylheptan-3-one* (**20**)

To a stirred solution of compound **2m** (0.885 g, 2.23 mmol) in MeOH (10 mL) was added Pd/C (45 mg, 10% *w*/*w*) in one portion. The resulting mixture was hydrogenated under 1 atmosphere of H_2_ at room temperature. After 2 h, the mixture was filtered by celite and concentrated to give the crude product, which was purified by flash chromatography on silica gel with EtOAc/*n*-hexane/ (1:2) to afford compound **19** (465 mg, 52% yield) as a colorless oil and compound **20** (206 mg, 23% yield) as a colorless oil.

Compound **19**: ^1^H-NMR (CDCl_3_) δ 6.83 (d, *J* = 8.0 Hz, 2H), 6.65 (d, *J* = 2.0 Hz, 2H), 6.63–6.61 (m, 2H), 5.51 (s, 2H), 3.87 (s, 6H), 2.76 (t, *J* = 7.0 Hz, 4H), 2.58 (t, *J* = 7.0 Hz, 4H), 1.29 (s, 6H); ^13^C-NMR (CDCl_3_) δ 208.9, 146.4, 143.9, 132.7, 120.9, 114.3, 114.9, 111.7, 62.4, 55.9, 40.5, 29.4, 21.0; HRMS [ESI]^+^ calculated for C_23_H_28_NaO_6_: 423.1784 [M + Na]^+^; found: 423.1780.

Compound **20**: ^1^H-NMR (CDCl_3_) δ 6.86–6.83 (m, 2H), 6.72–6.66 (m, 4H), 5.62–5.61 (br s, 2H), 3.89 (s, 3H), 3.87 (s, 3H), 3.71–3.69 (m, 1H), 2.90–2.71 (m, 5H), 2.61–2.51 (m, 2H), 1.71–1.55 (m, 2H), 1.13 (s, 3H), 1.08 (s, 3H); ^13^C-NMR (CDCl_3_) δ 216.5, 146.4, 143.9, 143.7, 133.9, 133.1, 120.9, 120.8, 114.4, 114.3, 111.2, 111.1, 55.9, 51.7, 40.3, 33.7, 32.6, 29.4, 21.7, 19.3; HRMS [ESI]^+^ calculated for C_23_H_30_NaO_6_: 425.4768 [M + Na]^+^; found: 425.4760.

#### 3.1.12. *1,7-bis(4-Hydroxy-3-methoxyphenyl)-4,4-dimethylheptane-3,5-diol* (**21**)

To a solution of compound **20** (110 mg, 0.27 mmol) in MeOH (3 mL) was added NaBH_4_ (30 mg, 0.81 mmol) at 0 °C. The resulting mixture was allowed to stir at room temperature for 2 h, and then H_2_O (3 mL) was added. The aqueous layer was separated and extracted with CH_2_Cl_2_ (3 × 6 mL). The combined organic extracts were washed brine, dried over MgSO_4_, filtered and concentrated to give the crude residue, which was purified by flash chromatography on silica gel with EtOAc/*n*-hexane/ (1:1) to afford compound **21** (34 mg, 31% yield) as a colorless oil. ^1^H-NMR (CDCl_3_) δ 6.86 (d, *J* = 8.0 Hz, 2H), 6.74–6.71 (m, 4H), 5.63 (s, 2H), 3.89 (s, 6H), 3.59–3.56 (m, 2H), 3.11 (s, 2H), 2.85–2.71 (m, 2H), 2.61–2.55 (m, 2H), 1.81–1.72 (m, 4H), 0.90 (s, 6H); ^13^C-NMR (CDCl_3_) δ 146.5, 143.7, 134.1, 134.0, 120.9, 114.3, 111.1, 78.7, 55.9, 40.3, 33.9, 32.8, 21.0; HRMS [ESI]^+^ calculated for C_23_H_32_NaO_6_: 427.4928 [M + Na]^+^; found: 427.4933.

### 3.2. Biological Assays

#### 3.2.1. In Vitro MTT [3-(4,5-Dimethylthiazaol-2-yl)-2,4-diphenyltetrazolium bromide] Assay

Cell viability was evaluated by measuring the reduction in MTT to yield blue formazan. Cells were cultured in 96-well plates, allowed to attach overnight, and then treated with compounds. After treatment, MTT solution (1 mg/mL) was added to each well, and plates were incubated for another 2 h. Medium was removed, blue formazan was dissolved in DMSO, and the absorbance was read at 570 nm by Multiskan Go spectrophotometer (Thermo Scientific, Madison, WI, USA).

#### 3.2.2. In Vivo Antitumor Activity Assay

Male nu/nu mice (5 weeks old) from National Laboratory Animal Center (Taipei, Taiwan) were maintained under the procedures and guidelines provided by the Institutional Animal Care and Use Committee of the National Health Research Institutes (Taipei, Taiwan). All experiments were supervised under the Institutional Animal Care and Use Committee, China Medical University, Taichung, Taiwan with a protocol number (CMUIACUC-2018-278). HCT-116 colon cancer cells (5 × 10^6^ cells per mouse) were suspended in 0.1 mL of Matrigel solution (50% *v*/*v* Matrigel in PBS) and inoculated into the mammary fat pads of the mice. When the tumor size reached 100 mm^3^, the tumor-bearing mice were randomly divided into four groups for treating with vehicle (5% ethanol and 10% tween 80 in 85% saline, 0.1 mL) and **2** (p.o. 50, 100 and 150 mg/kg/day body weight) respectively. Compounds was administered via oral route daily for 30 days. 5-Fluorouracil-treated group (i.p. 30 mg/kg) is the positive control. Tumor size and mouse body weight were measured once every 3 days, and tumor volume (mm^3^) was calculated using the equation: length × (width)^2^ × 0.5. At the end of experiments, mice were sacrificed.

#### 3.2.3. The Preliminary Pharmacokinetic Evaluation of 2 in Rat

##### Drug administration and blood collection

Male Sparague-Dawley rat (420 g) from National Laboratory Animal Center, Taipei, Taiwan were maintained under the regulations of the Institutional Animal Care and Use Committee of the National Health Research Institutes, Taiwan. The animal studies were supervised under the Institutional Animal Care and Use Committee, China Medical University, Taichung, Taiwan with a protocol number (CMUIACUC-2018-278). Rat was fasted for 16 h before dosing. Compound **2** was dissolved in aqueous solution (5% ethanol and 10% Tween80 in 85% saline) and administrated via oral gavage at a dosage of 100 mg/kg. Blood samples were collected at selected time points (5, 10, 20, 30, 60, 120, 240, 360, 480 min) post dose and centrifuged at 10,000× *g* for 15 min to provide serum samples, which were stored at −80 °C until subsequent sample extraction.

#### 3.2.4. Analysis of **2**, **2m**, **18, 19, 20, 21, 22** and **23** in Serum

Methods for the preparation of **2**, **2m**, **18**, **19**, **20** and **21** serum sample are delineated as follows. 50 μL of acetate buffer (pH 5.0), 50 μL of ascorbic acid (200 mg/mL), and 50 μL of 0.1 N HCl solution was added into 100 μL of serum which was collected at different time points). The resulting mixture was partitioned with 250 μL of ethyl acetate (containing 5.0 μg/mL of butyl paraben as the internal standard). After centrifuging at 10,000× *g* for 15 min, the upper organic layer was separated and dried under nitrogen gas to provide the crude sample, which was diluted with an appropriate volume of acetonitrile for LC-MS analysis.

The amount of **22** and **23** were analyzed by the serum samples before and after treatment with enzyme solution. The procedure for enzyme treatment is shown as follows. 100 μL of serum was treated with enzyme solution (50 μL; containing 1000 units/mL of sulfatase and 39861 units/mL of β-glucuronidase in pH 5.0 acetate buffer), 50 μL of 0.1 N HCl solution and 50 μL of ascorbic acid (200 mg/mL) in the light protected tubes at 37 °C. For optimal hydrolysis efficiency, the incubation time was decided as 2 h based on a previous study [35]. The mixture was then partitioned with 250 μL of ethyl acetate (containing 5.0 μg/mL of butyl paraben as the internal standard). After centrifuging at 10,000× *g* for 15 min, the upper organic layer was separated and dried under nitrogen gas to provide the crude sample, which was diluted with an appropriate volume of acetonitrile for LC analysis.

#### 3.2.5. LC-MS Analysis Method

All samples obtained from in vivo pharmacokinetic studies were assayed using LC-MS for the analysis of **2** and the corresponding metabolites. The Waters ACQUITY UPLC I-Class system (Waters Corp., Milford, MA, USA) was utilized. System control and all of the mass spectrometry data were acquired and analyzed using UNIFI software (Waters Corp.). For sample separation, the LC was equipped with an Inertsil ph-3 (50 × 2.1 mm, 2 μm particle size) RPLC column (GL Sciences Inc. Tokyo, Japan) in which the column temperature was held at 35 °C, injection volume was 7.5 μL, and the flow rate of the mobile phase was 200 μL/min. The elution started from 50% mobile phase A (ultrapure water + 0.1% formic acid) and 50% mobile phase B (100% methanol + 0.1% formic acid), held at 50% B for 0.5 min, raised to 95% B in 5.5 min, held at 95% B for 1 min, and then lowered to 50% B in 1 min. Before sample injection, the column was equilibrated by pumping 50% B for 4 min. The analysis of target compounds for all samples was performed by using a Waters Vion IMS QTof mass spectrometer. Data were acquired in the electrospray ionization (ESI) positive ion MS^E^ mode with range of *m*/*z* 100–1000 and 0.5 s scan time. Parameters were set as capillary voltage of 2.5 kV, source temperature of 100 °C, desolvation temperature at 250 °C, cone gas maintained at 10 L/h, desolvation gas maintained at 600 L/h. The acquired m/z and isotope pattern were processed by UNIFI software to illustrate chromatogram, and the amounts of compound was calculated with the integrated peak area of signals.

## 4. Conclusions

Here, structural modifications at the C-4 position of compound **1**, a 2,2-bis(hydroxymethyl)-propionate curcumin prodrug, with polar and nonpolar functional groups were performed to form a series of 4,4-disubstituted analogs of **1** and **DMC**. The in vitro anti-proliferative assay of these derivatives elucidated an obvious SAR in which the enhancement of polarity on C-4 resulted in the erosion of anticancer activity. The ester prodrug characteristics of **2**–**6** motivated us to synthesize their hydrolysis compounds **2m**–**6m**, **15**, and **16**, whose anticancer activities were highly dependent on the volume and length of the C-4 alkyl chains. The preliminary pharmacokinetic evaluations indicated that the esterase-mediated hydrolysis of **2** proceeded smoothly to produce **2m**, which in turn was reduced to **18** and **19** as major phase I metabolites. The phase II metabolic pathway was confirmed by the detection of glucuronide **22** and sulfate **23**. However, it is still possible that reduction precedes ester hydrolysis to provide the hydrogenated counterparts of **2**, which possess novel chemical skeletons with unknown biological activity.

Our preliminary pharmacokinetic results also shed light on future directions for the structural modification of the original compounds. Additional modifications have been scheduled to reduce the high propensity towards ester hydrolysis by converting the methyl of 2,2-bis(hydroxymethyl)-propionate group into more bulky alkyl groups. Pharmacokinetic studies have been designed to identify, biologically evaluate, and quantify the possible metabolites. Apart from this, compound **2** and **2m** may behave like **1** which acts on multiple targets and signaling pathways. The differences between the mechanism of action of **2**, **2m** and **1** are not definitively understood. Currently, we are actively investigating the mechanism of action and pharmacokinetic parameters of **2** and **2m**, including T_1/2_, AUC, and C_max_, and the results will be reported in due course. The animal study showed that compound **2** inhibits tumor growth by 45% at a dose of 100 mg/kg in HCT-116 xenograft nude mice. Thus, twice-a-day (BID) dosing formulations for achieving higher therapeutic efficacy at lower doses should be considered in further development of title curcuminoid derivatives as anticancer drug candidates.

## Figures and Tables

**Table 1:**
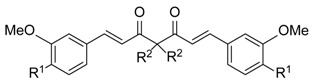
The anti-proliferative effects of curcumin derivatives against MDA-MB-231 and HCT-116 cell lines.

Compound	R^1^	R^2^	IC_50_^a^ (M)/ 48 h	
			MDA-MB-231	HCT-116
**Curcumin**	**OH**	H	22.05 ± 2.97	26.33 ± 1.38
**1**	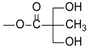	H	3.06 ± 0.18	6.25 ± 0.60
**2**	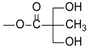	methyl	2.25 ± 0.10	3.10 ± 0.29
**3**	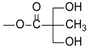	ethyl	3.02 ± 0.15	1.93 ± 0.01
**4**	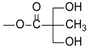	benzyl	2.45 ± 0.24	7.66 ± 0.61
**5**	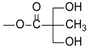	propargyl	5.87 ± 0.10	5.81 ± 0.23
**6**	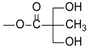	allyl	3.13 ± 0.54	0.92 ± 0.06
**9**	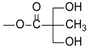	CH_2_OH	>100	>100
**10**	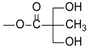	CH_2_OAc	6.55 ± 0.04	6.53 ± 0.01
**11**	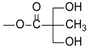	CH_2_OMe	84.92 ± 1.67	69.08 ± 3.11
**12**	OCH_3_	CH_2_OH	53.50 ± 3.45	58.00 ± 0.60
**14**	OCH_3_	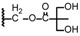	57.43 ± 2.06	40.10 ± 1.61
**2m**	OH	methyl	4.17 ± 0.15	2.17 ± 0.16
**3m**	OH	ethyl	9.12 ± 0.40	8.18 ± 0.57
**4m**	OH	benzyl	36.52 ± 1.66	9.40 ± 0.02
**5m**	OH	propargyl	3.10 ± 0.15	1.38 ± 0.06
**6m**	OH	allyl	6.88 ± 0.36	4.40 ± 0.53
**15**	OH	propyl	18.17 ± 0.53	10.73 ± 1.63
**16**	OH	butyl	>100	67.00 ± 3.18

^a^ All data presented are means from at least three experiments with standard deviations of the value quoted.

## Supplementary Materials

The following are available online. **Scheme S1**: PLE mediated hydrolysis of curcumin 2,2-bis(hydroxymethyl)propionate **1**. **Figure S1**: The HPLC analysis of compound **18**, **19**, **20**, **21**, **22** and **23**. Copies of ^1^H and ^13^C NMR spectra of the new products.
