# Supplementary material for: Synthesis, Anticancer Activity, and Preliminary Pharmacokinetic Evaluation of 4,4-Disubstituted Curcuminoid 2,2-bis(Hydroxymethyl)Propionate Derivatives
Source: Molecules. 2020 Jan 22;25(3):479. doi: 10.3390/molecules25030479 (PMC7037731; doi:10.3390/molecules25030479)
Supplement: Supplementary file 1 [file molecules-25-00479-s001.pdf]

Supporting Information

Synthesis, anticancer activity, and preliminary pharmacokinetic evaluation of 4,4-disubstituted curcuminoid 2,2-bis(hydroxymethyl)propionate derivatives

Der-Yen Lee, Yu-Chi Hou, Jai-Sing Yang, Hui-Yi Lin, Tsu-Yuan Chang, Kuo-Hsiung Lee, Sheng-Chu Kuo, Min-Tsang Hsieh

Porcine liver esterase mediated hydrolysis of curcumin 2,2-bis(hydroxymethyl)propionate 1

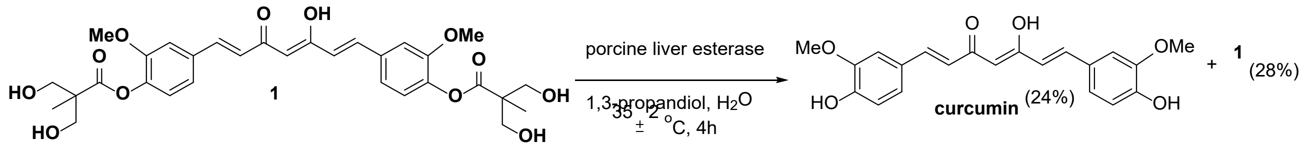

To a stirred solution of compound **1** (0.200 g, 0.29 mmol) in 1,3-propandiol/water (ratio = 1:3, 4mL) was added porcine liver esterase (10 mg, lyophilized powder, ≥15 units/mg solid) in one portion. The resulting mixture was stirred at 35 ± 2 °C for 4 hours under air atmosphere. H_2O (6 mL) was added to quench the reaction. The aqueous layer was separated and extracted with CH_2Cl_2 (3 × 6 mL). The combined organic extracts were washed with brine, dried over $MgSO_4$, filtered and concentrated to give the crude product, which was then purified by flash chromatography on silica gel with EtOAc/*n*-hexane/CH_2Cl_2 (1:2:1) to afford curcumin (0.026 g, 24% yield) as an yellow soild and recover compound **1** (0.056 g, 28% yield).

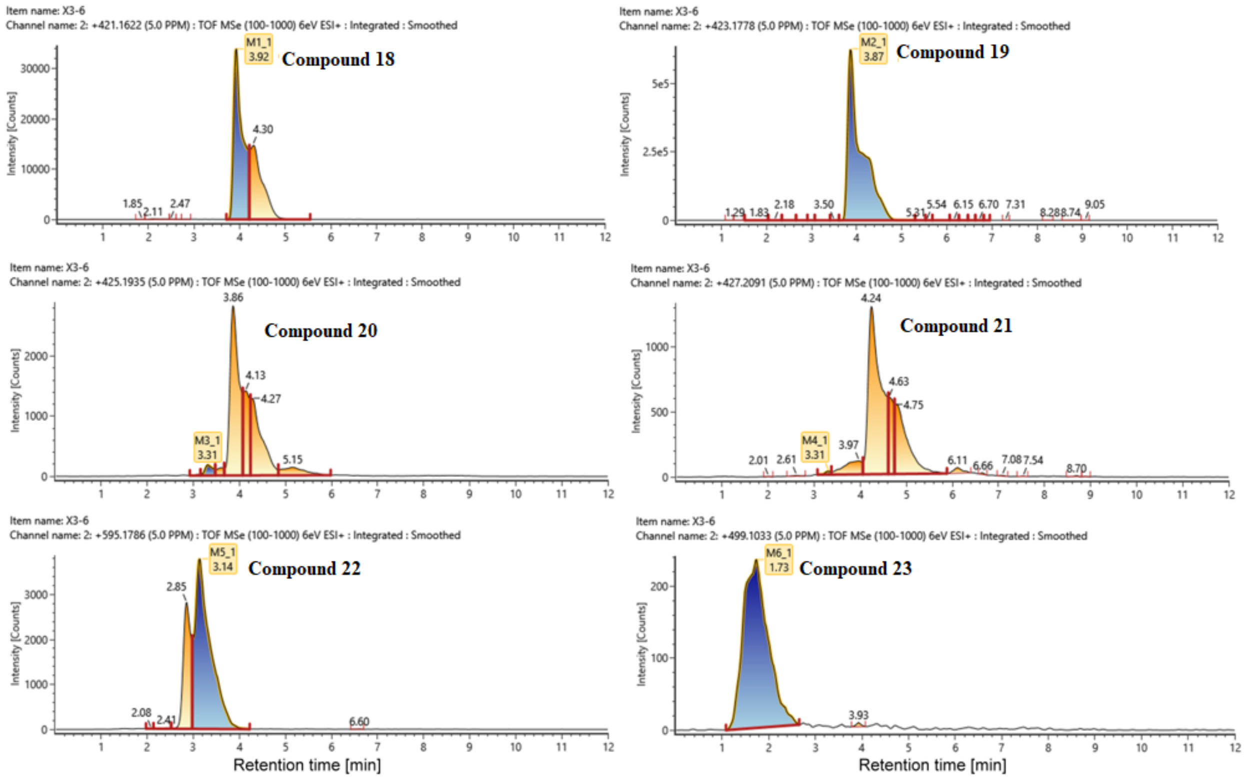

Figure S1. The analysis of compound **18**, **19**, **20**, **21**, **22** and **23** was performed by Waters Vion IMS QTof mass spectrometer. The acquired m/z and isotope pattern were processed by UNIFI software to illustrate chromatogram. The m/z values of the parent ions were set to 421.1622 for **18**, 423.1778 for **19**, 425.1935 for **20**, 427.2091 for **21**, 595.1786 for **22** and 499.1033 for **23**, respectively.

The NMR sepctrums of newly synthesized compounds

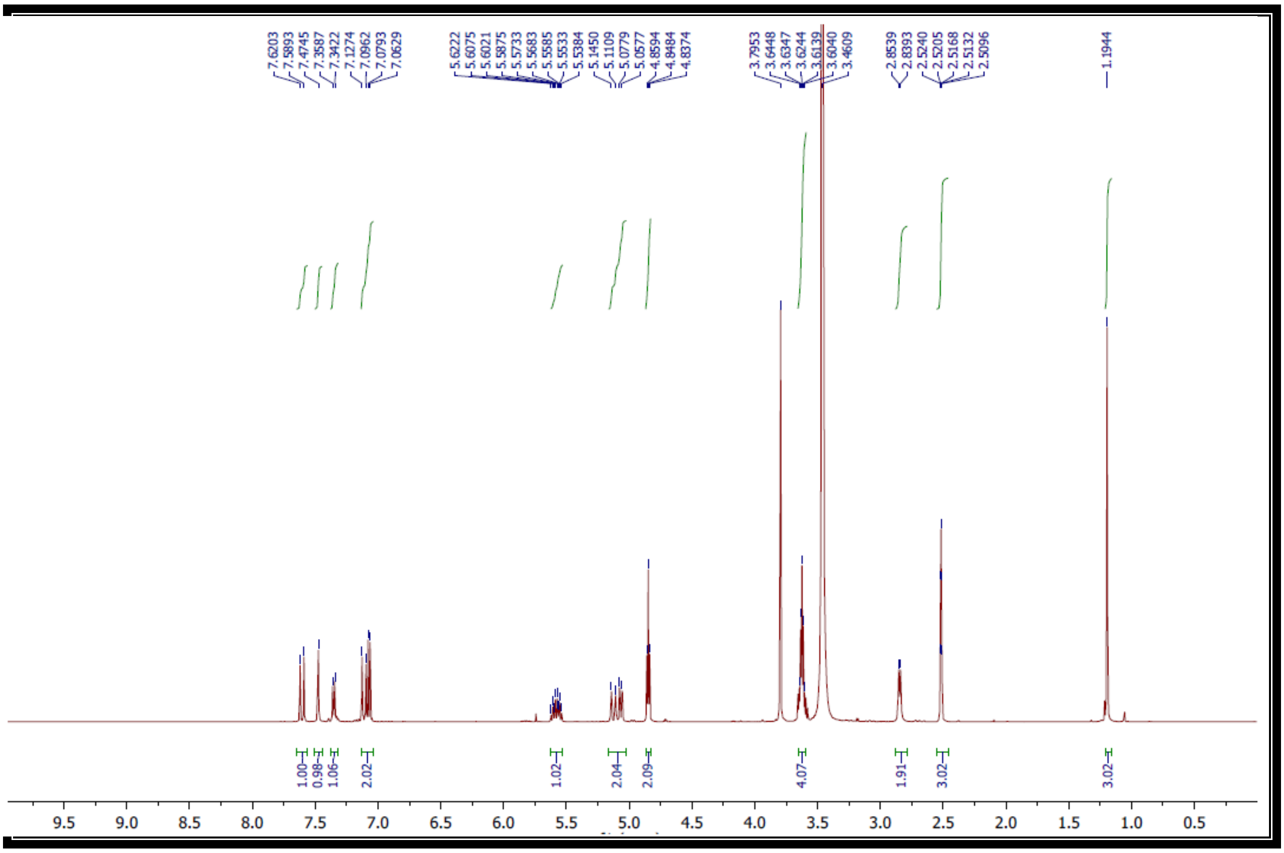

^{1}H spectrum of **6**

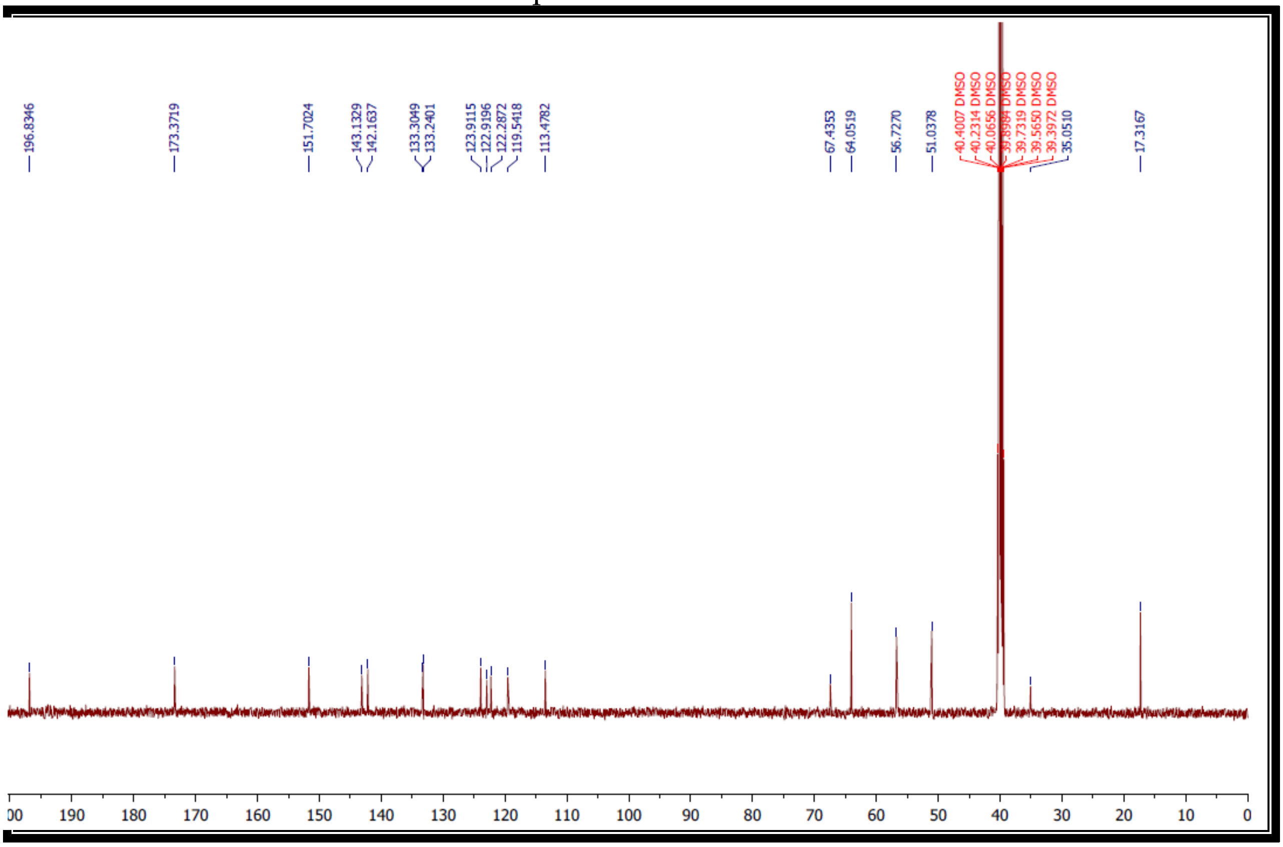

^{13}C spectrum of **6**

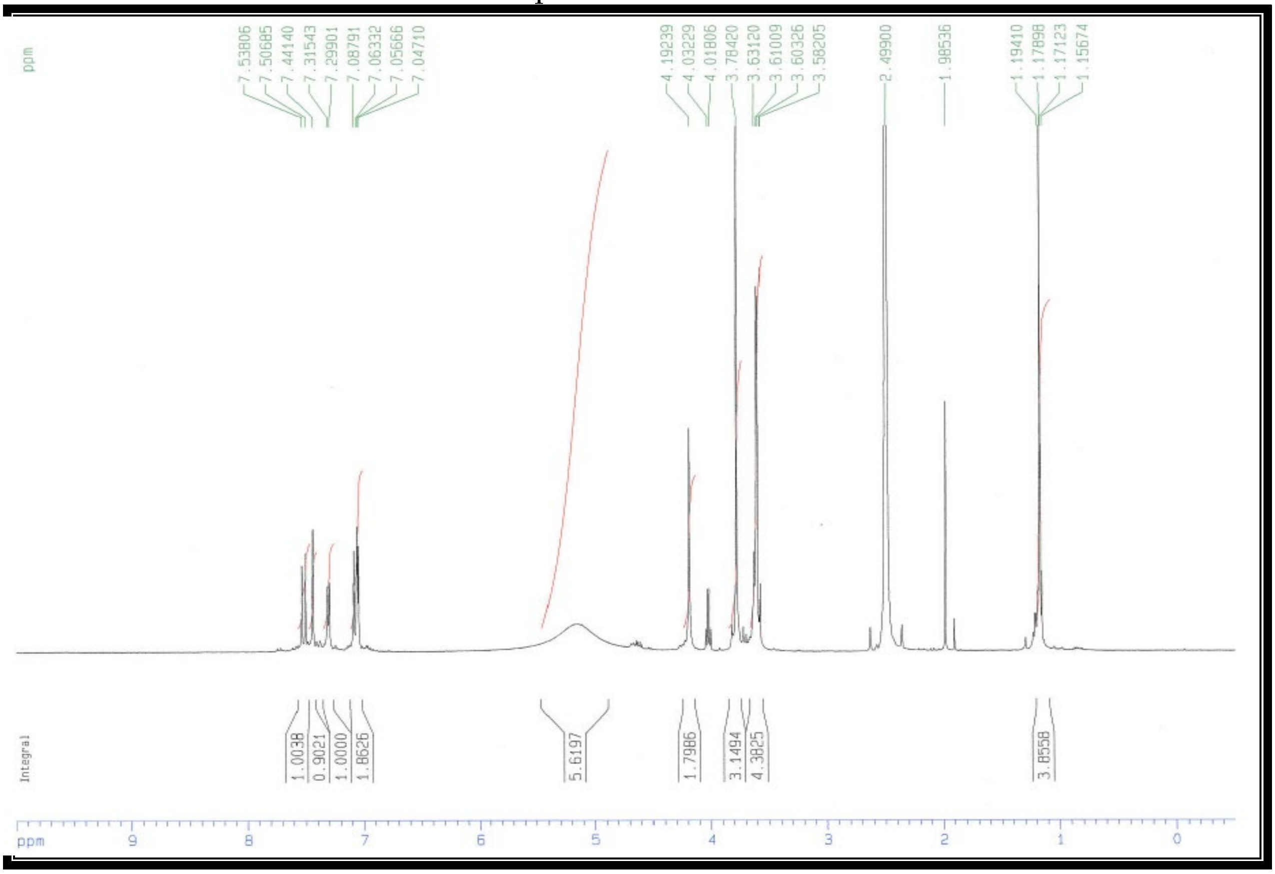

^{1}H spectrum of **9**

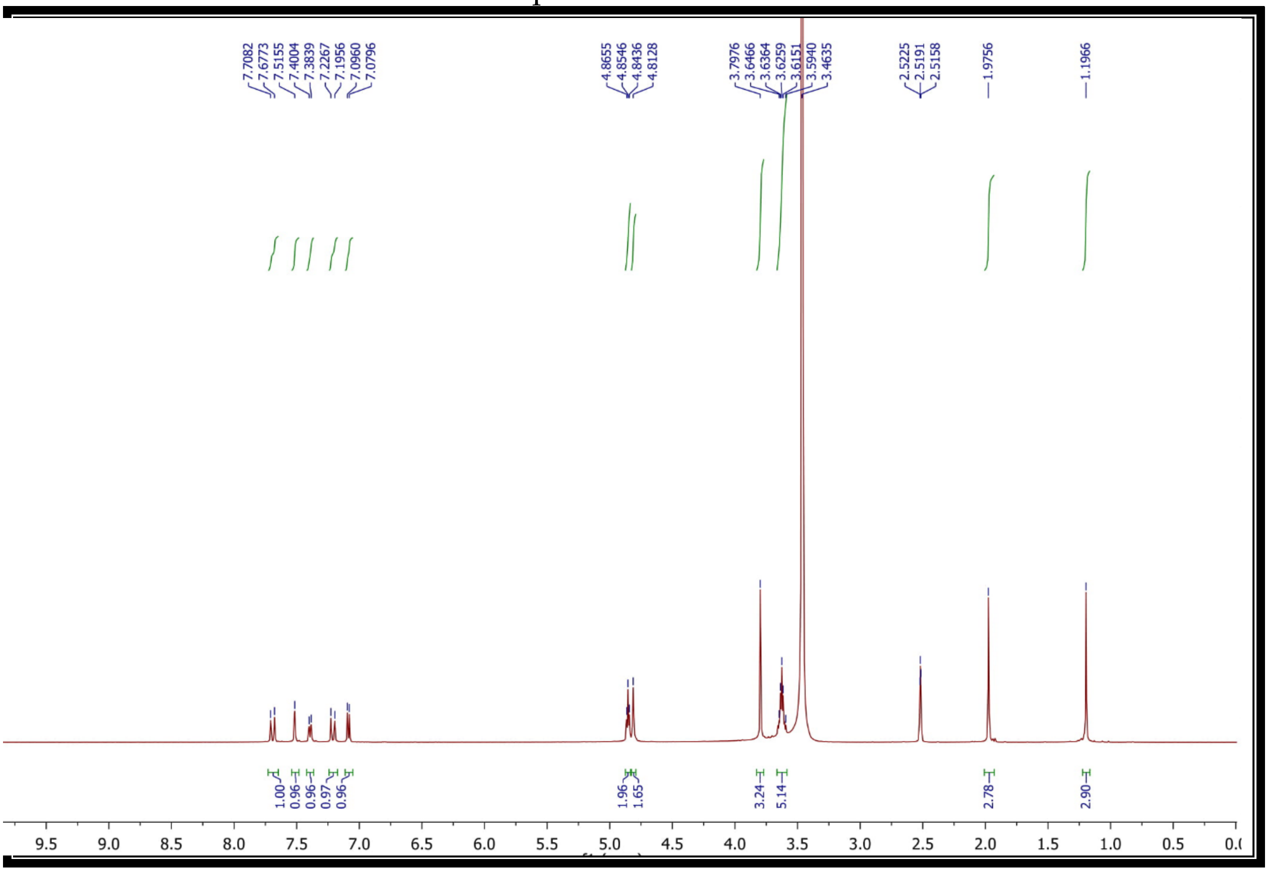

^{1}H spectrum of **10**

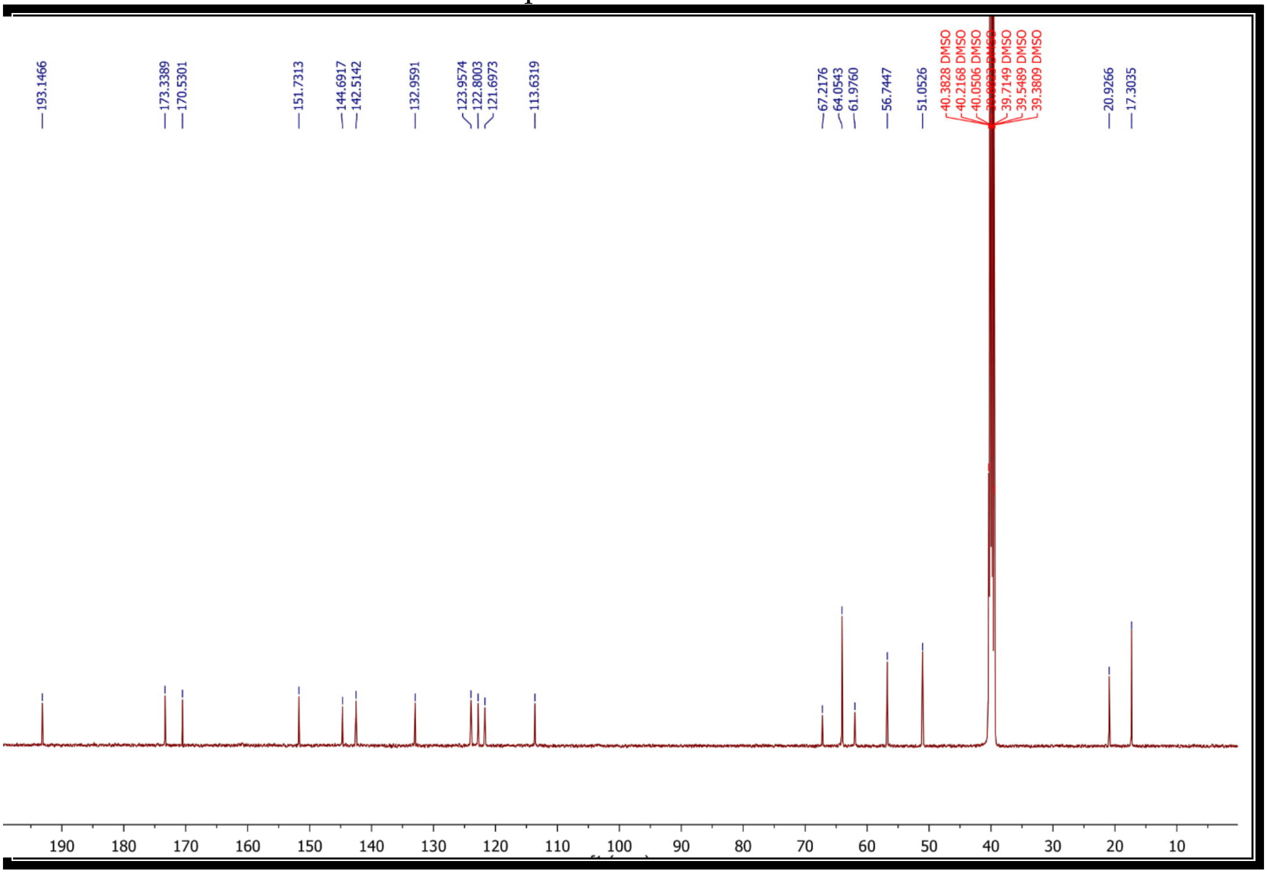

^{13}C spectrum of **10**

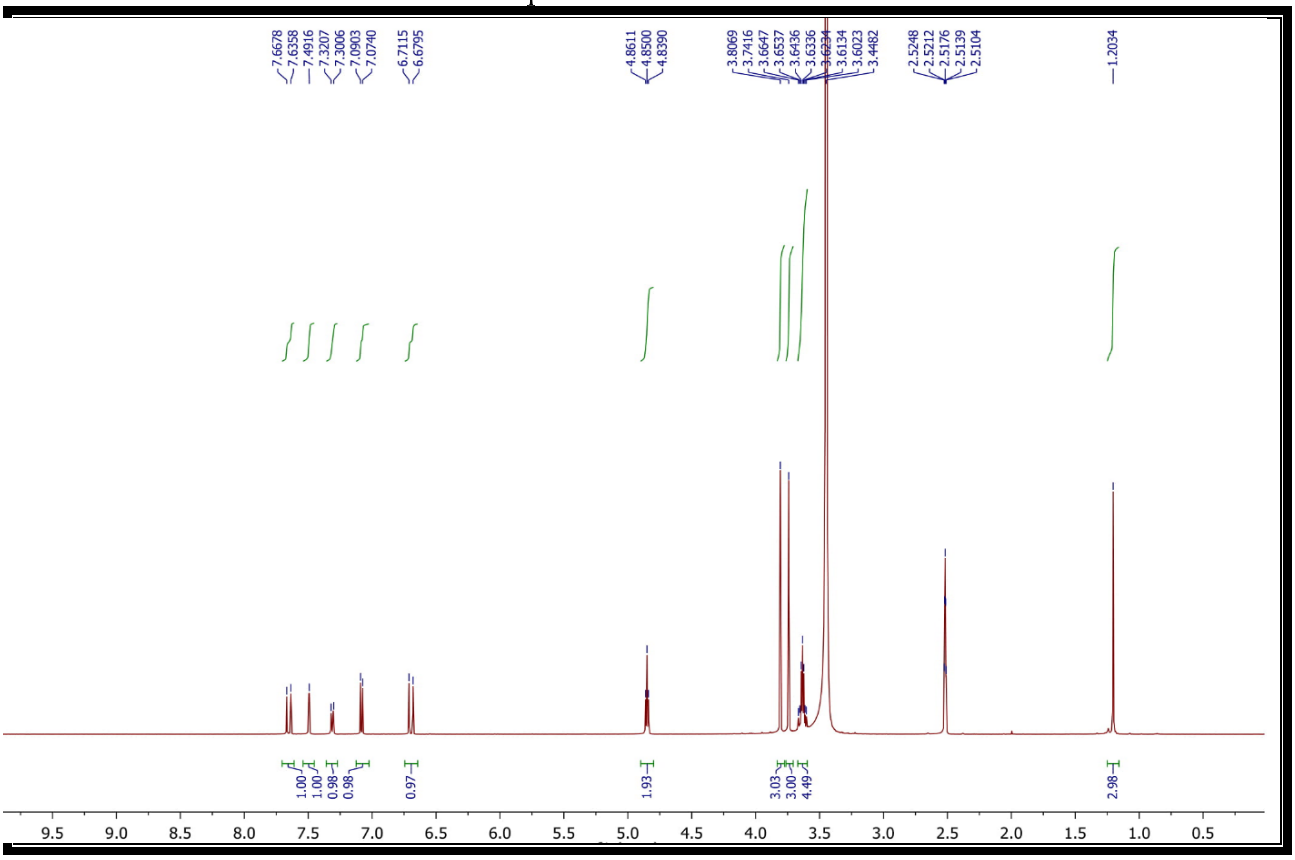

^{1}H spectrum of **11**

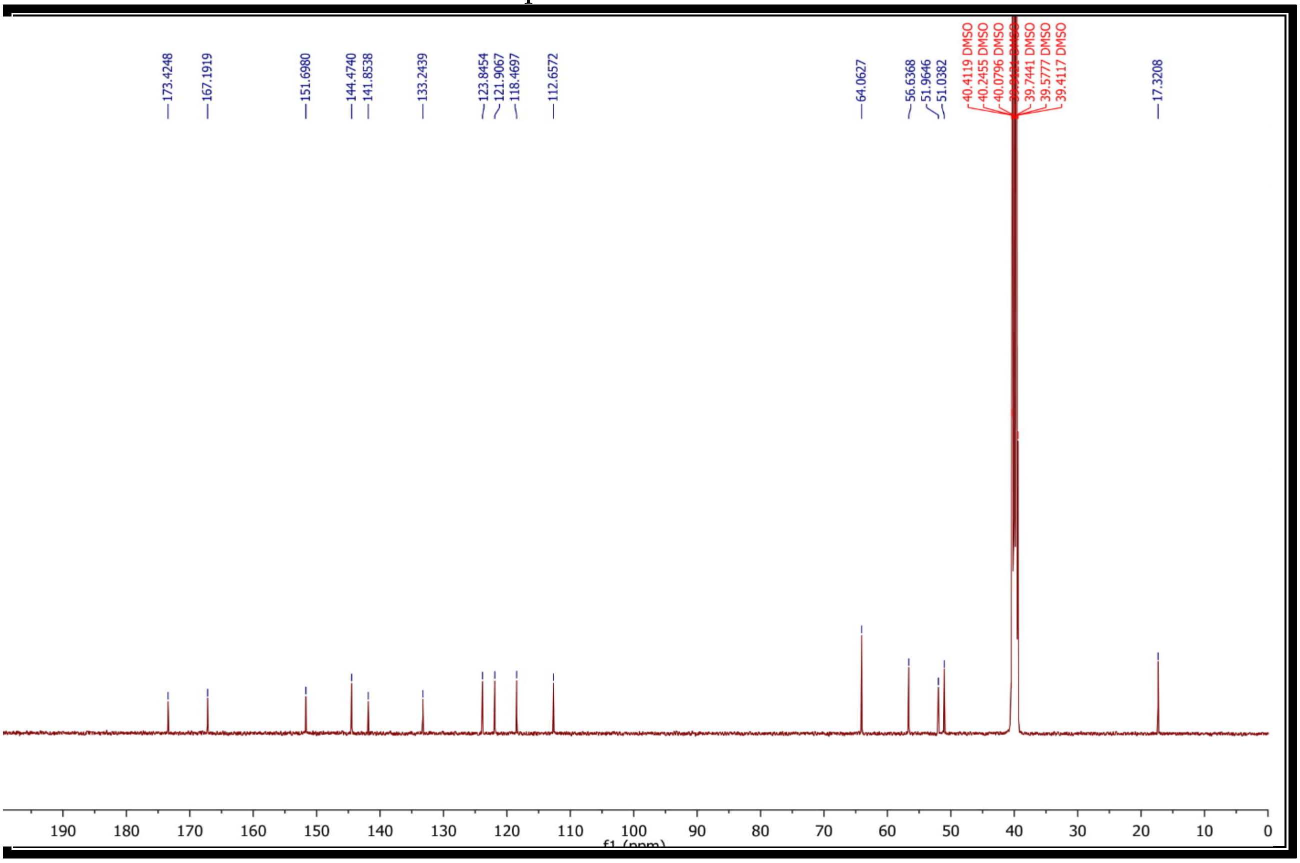

^{13}C spectrum of **11**

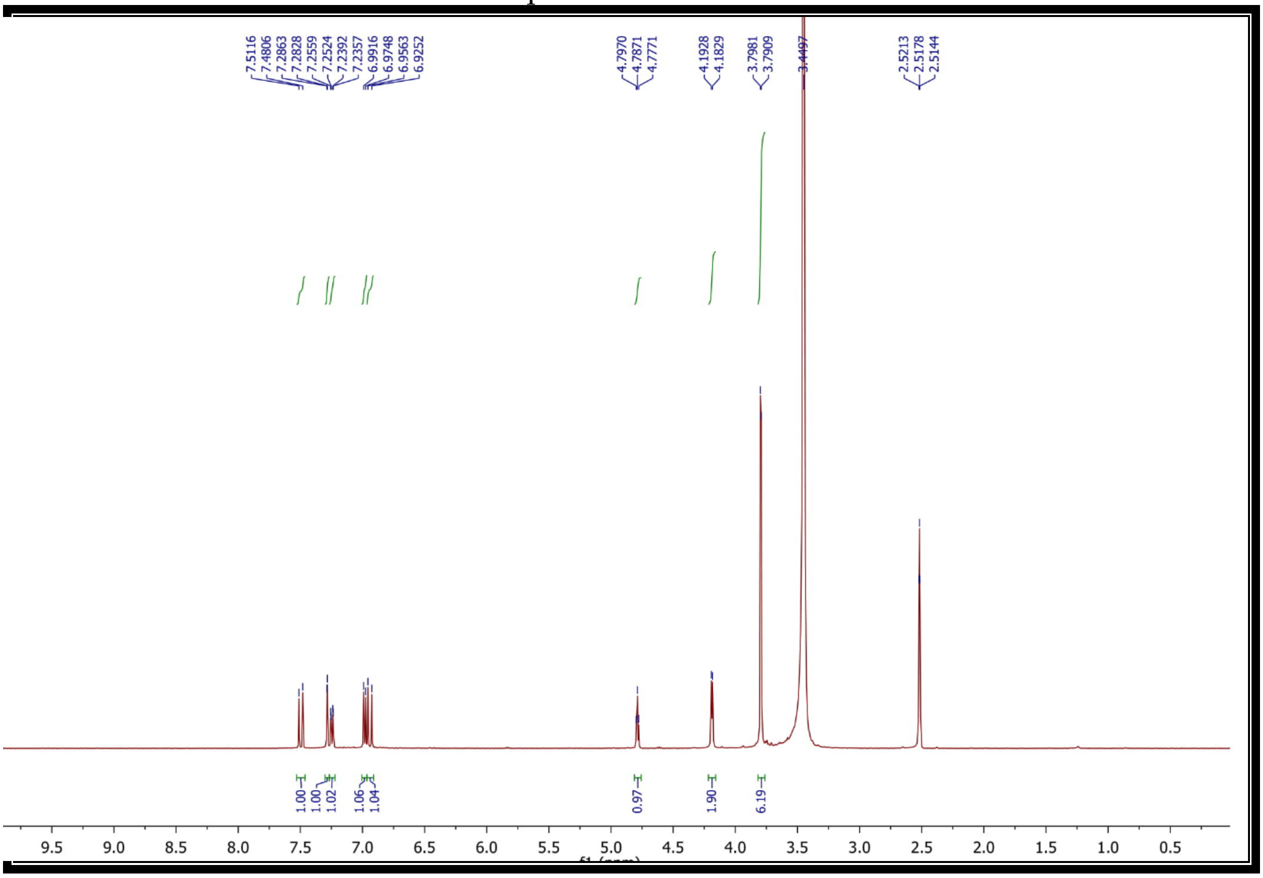

¹H spectrum of **12**

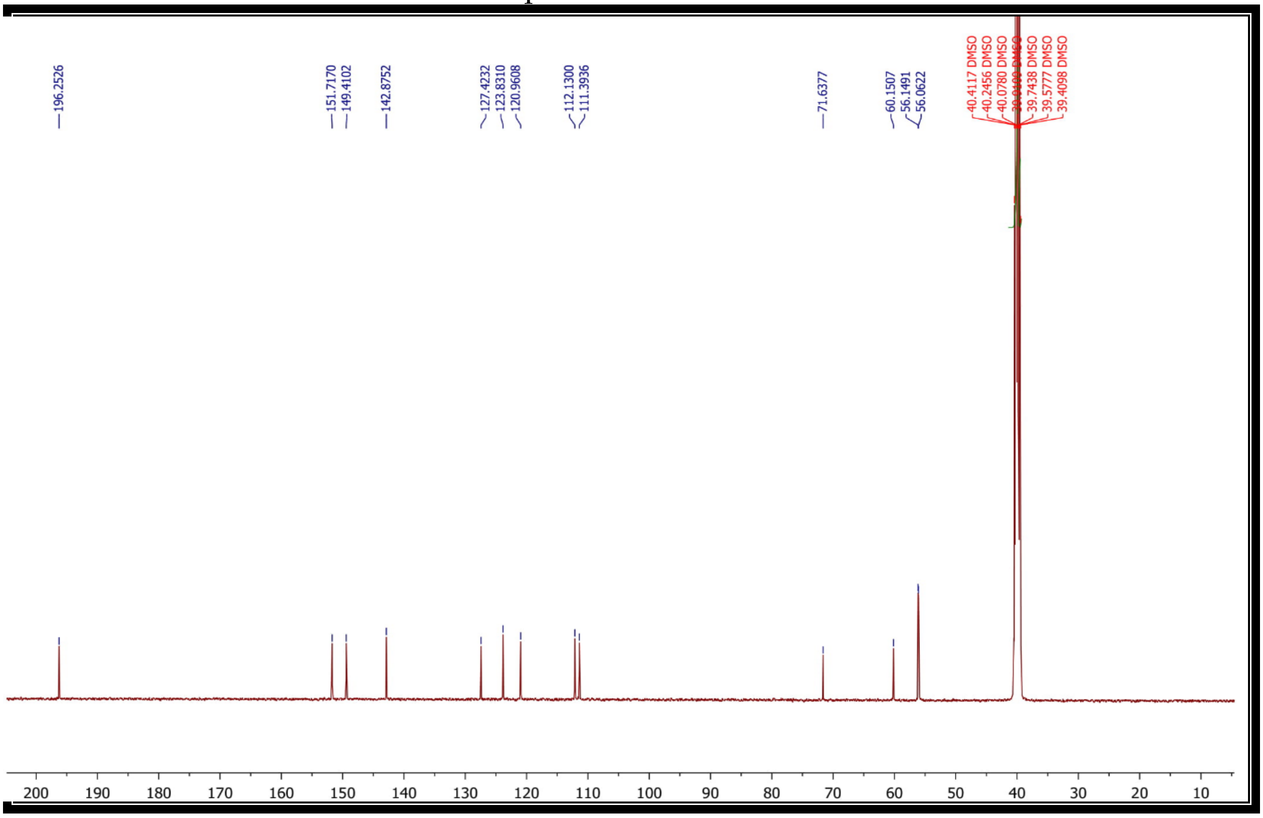

^{13}C spectrum of **12**

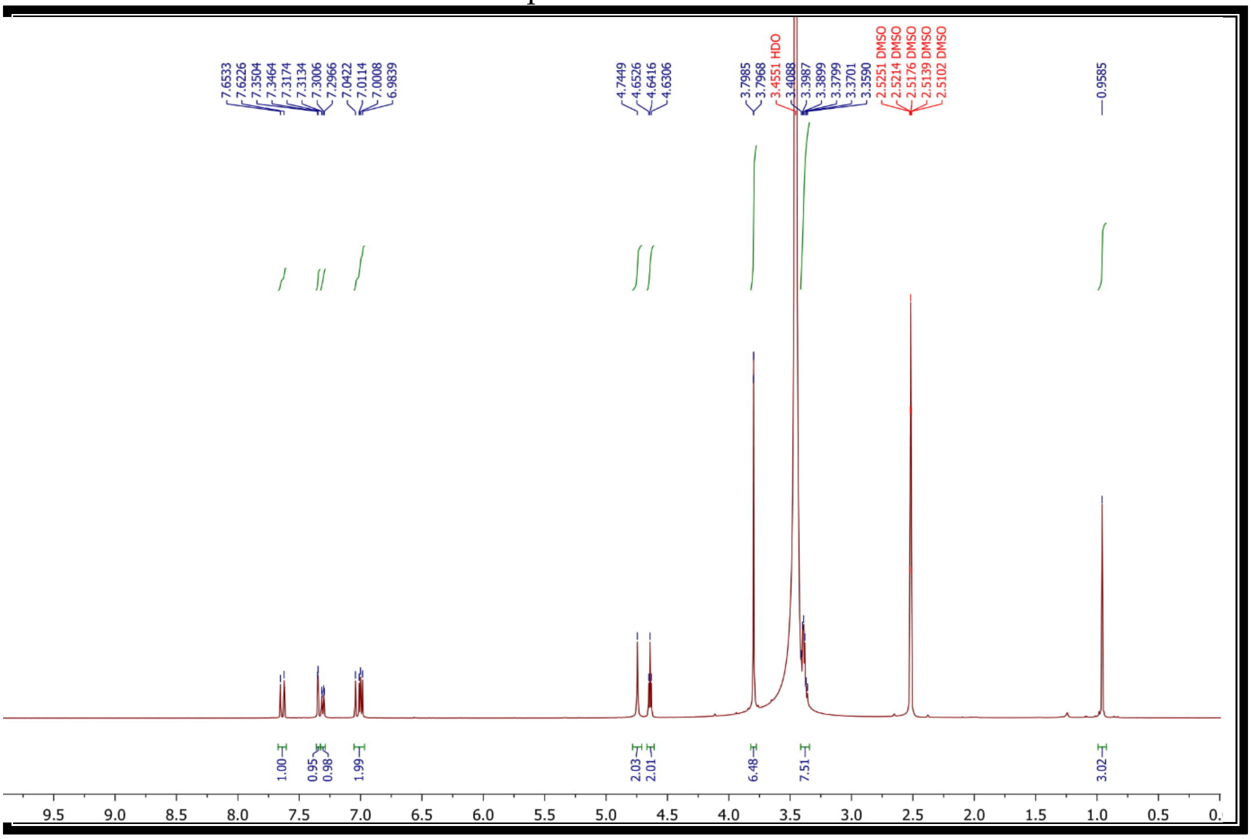

¹H spectrum of **14**

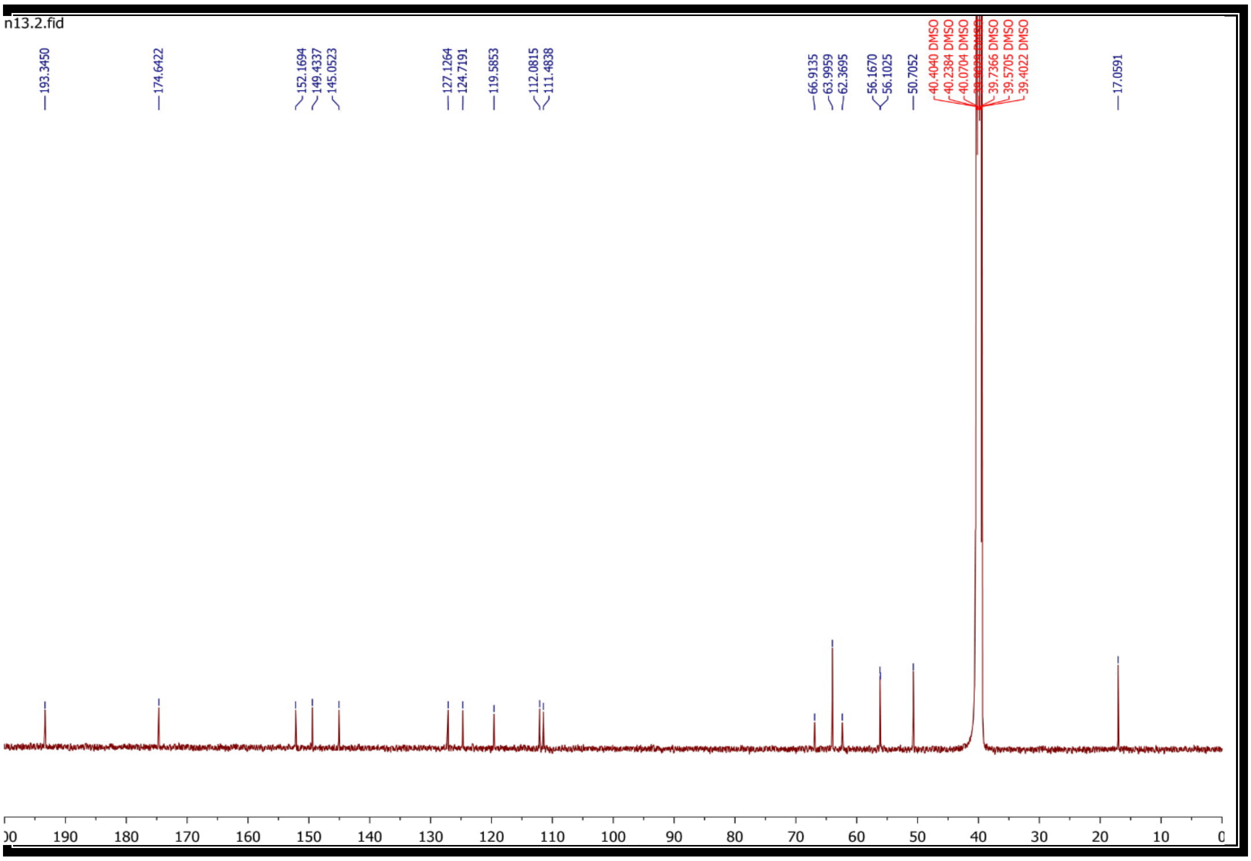

^{13}C spectrum of **14**

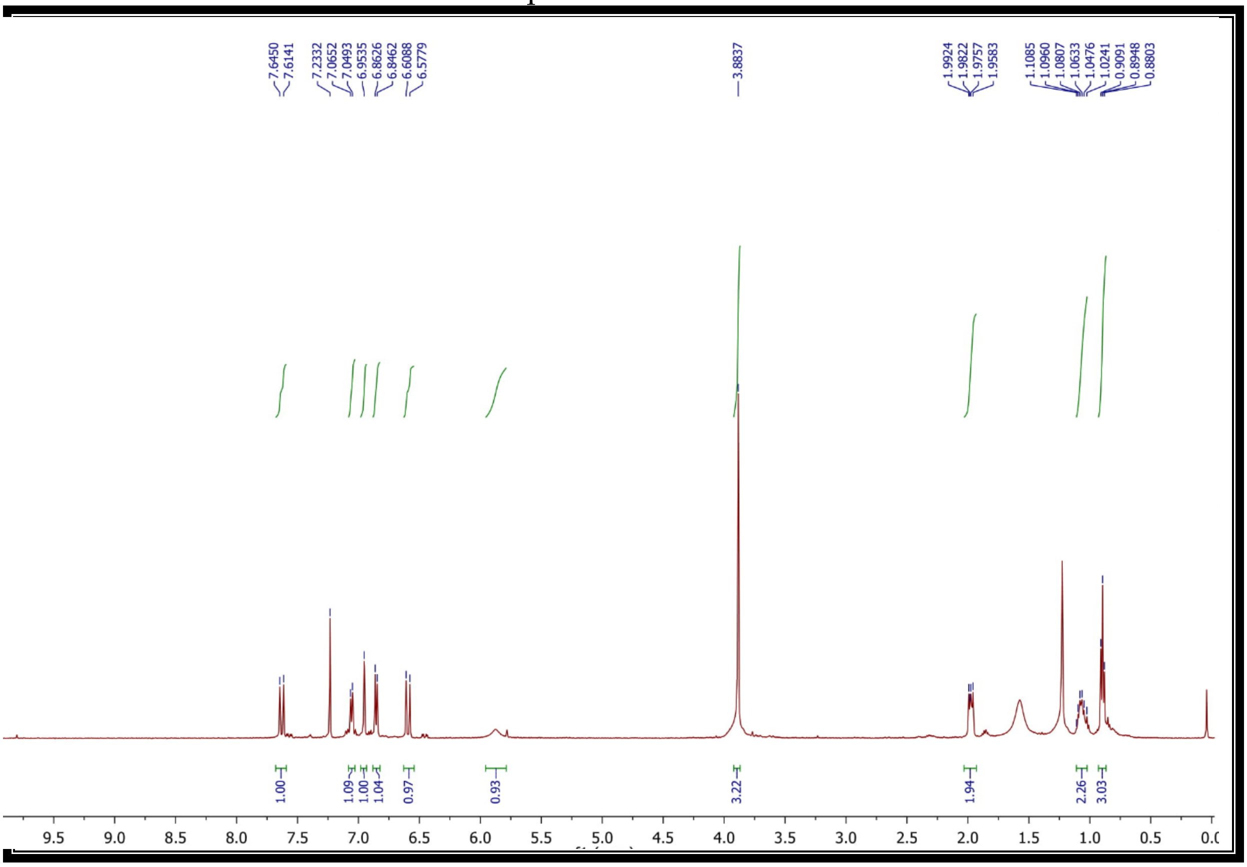

^{1}H spectrum of **15**

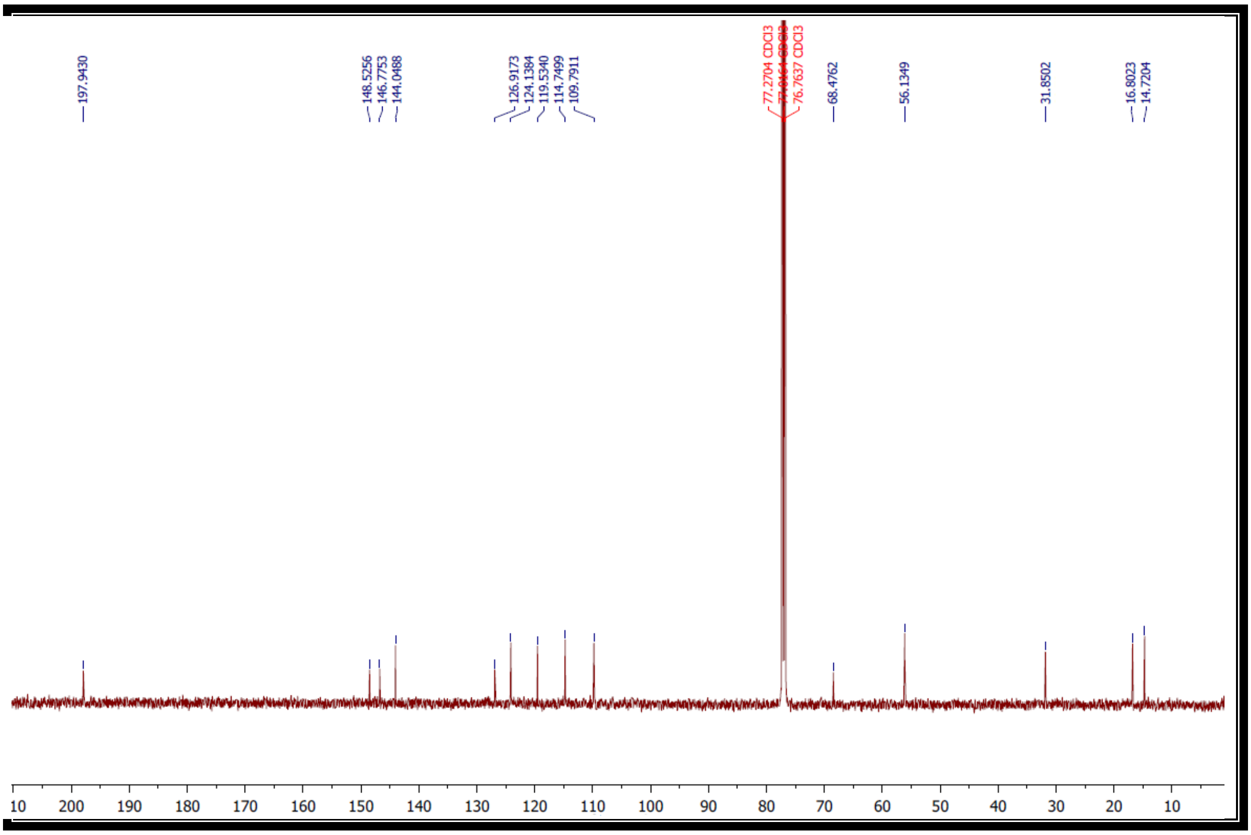

^{13}C spectrum of **15**

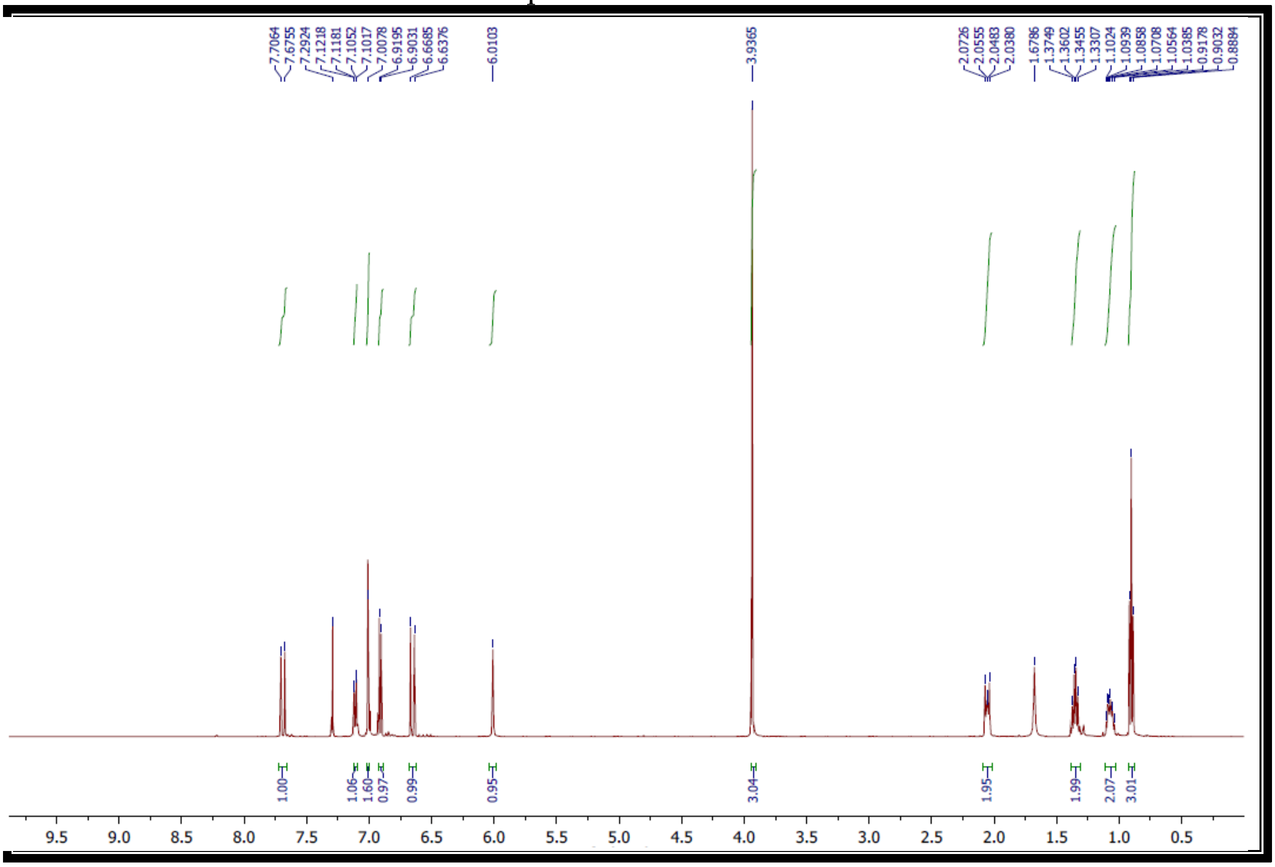

^{1}H spectrum of **16**

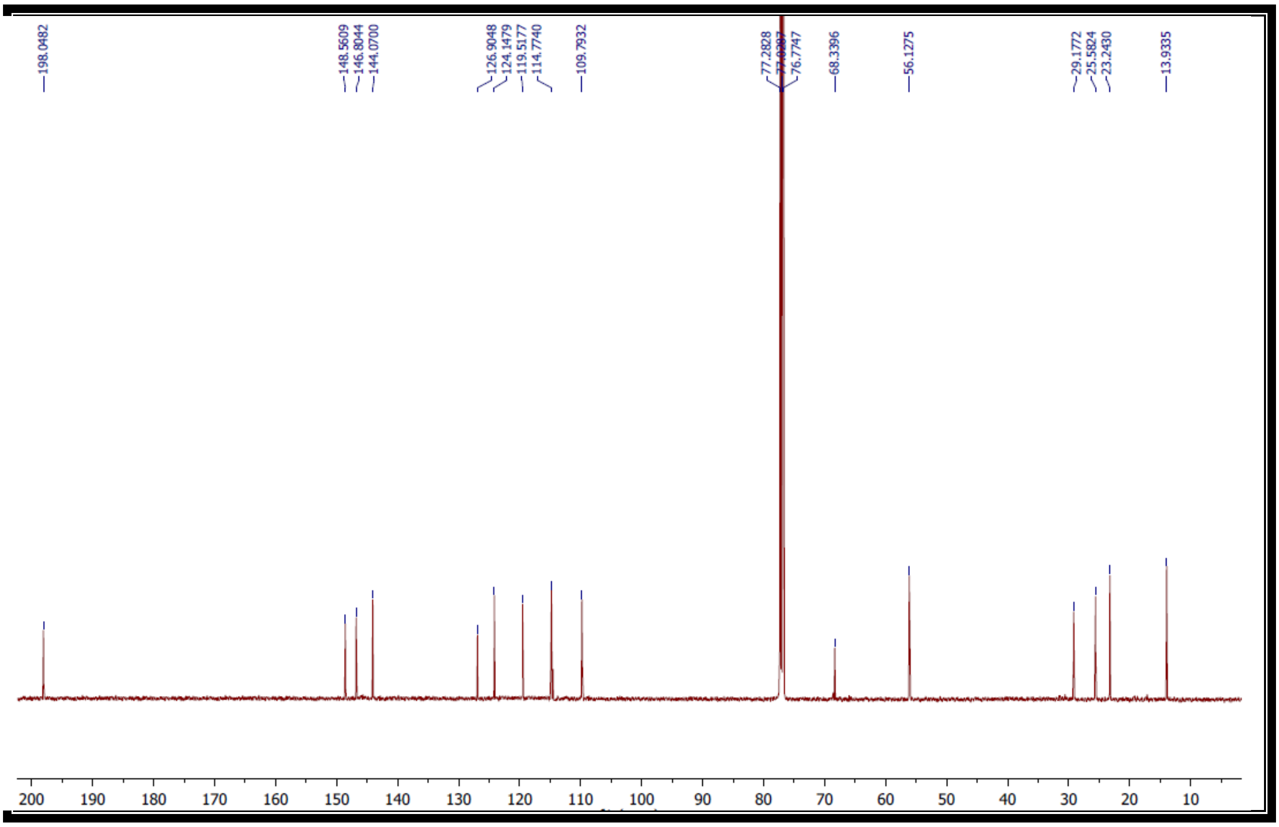

^{13}C spectrum of **16**

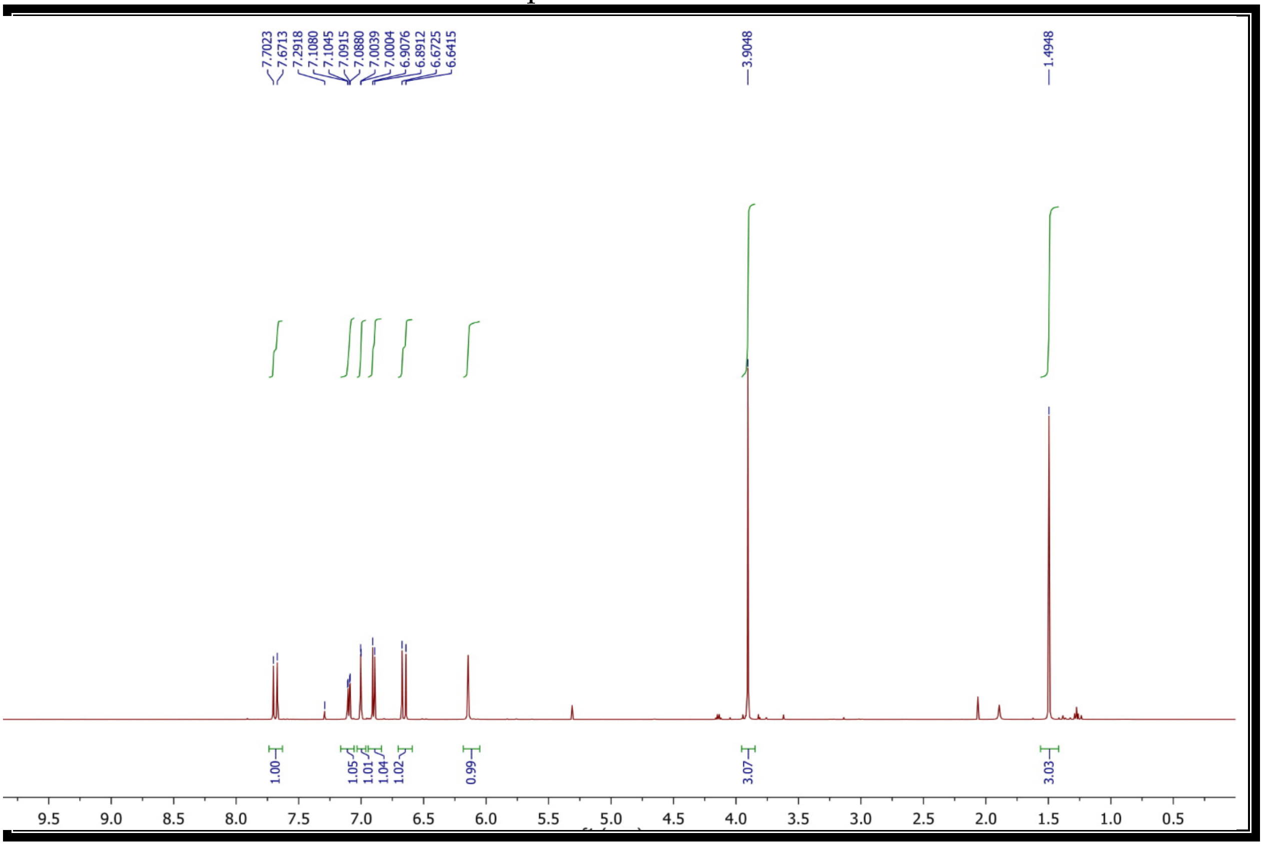

^{1}H spectrum of **2m**

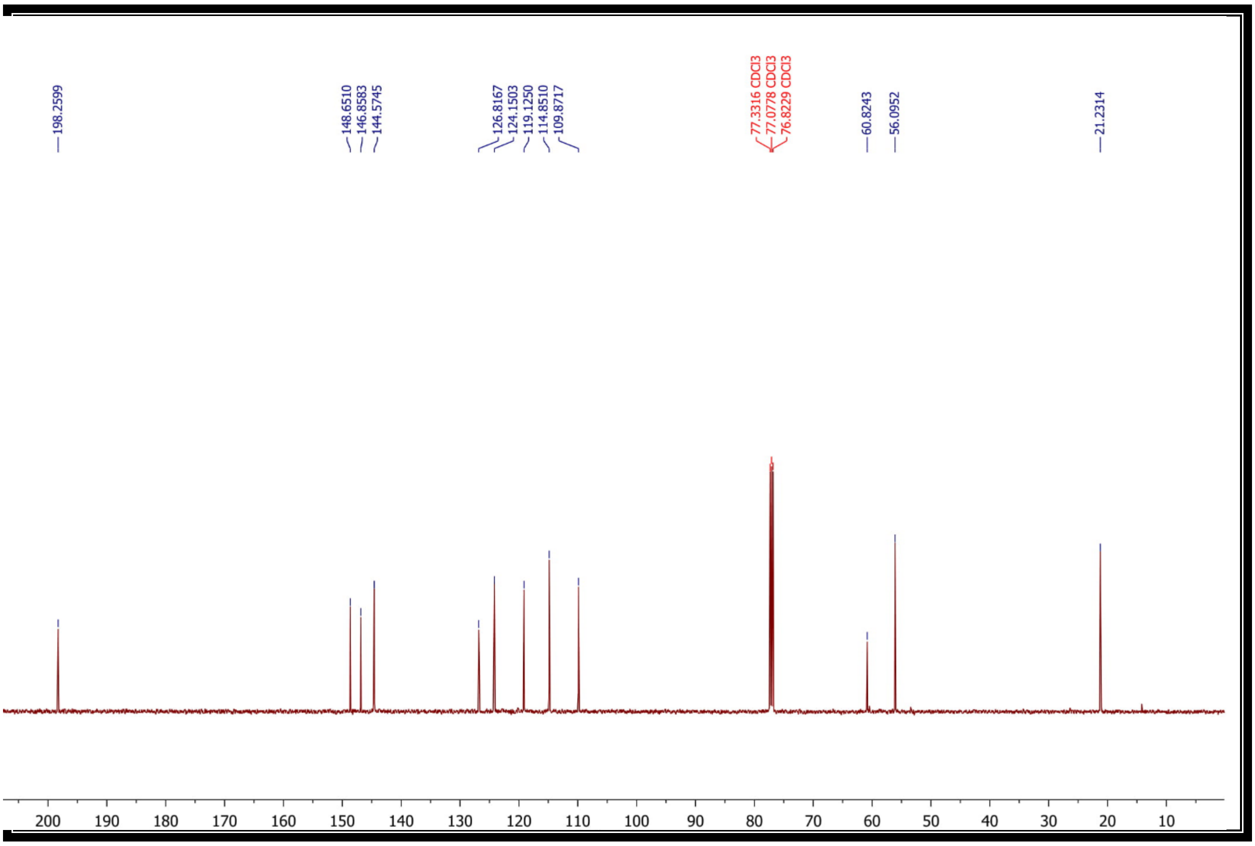

^{13}C spectrum of **2m**

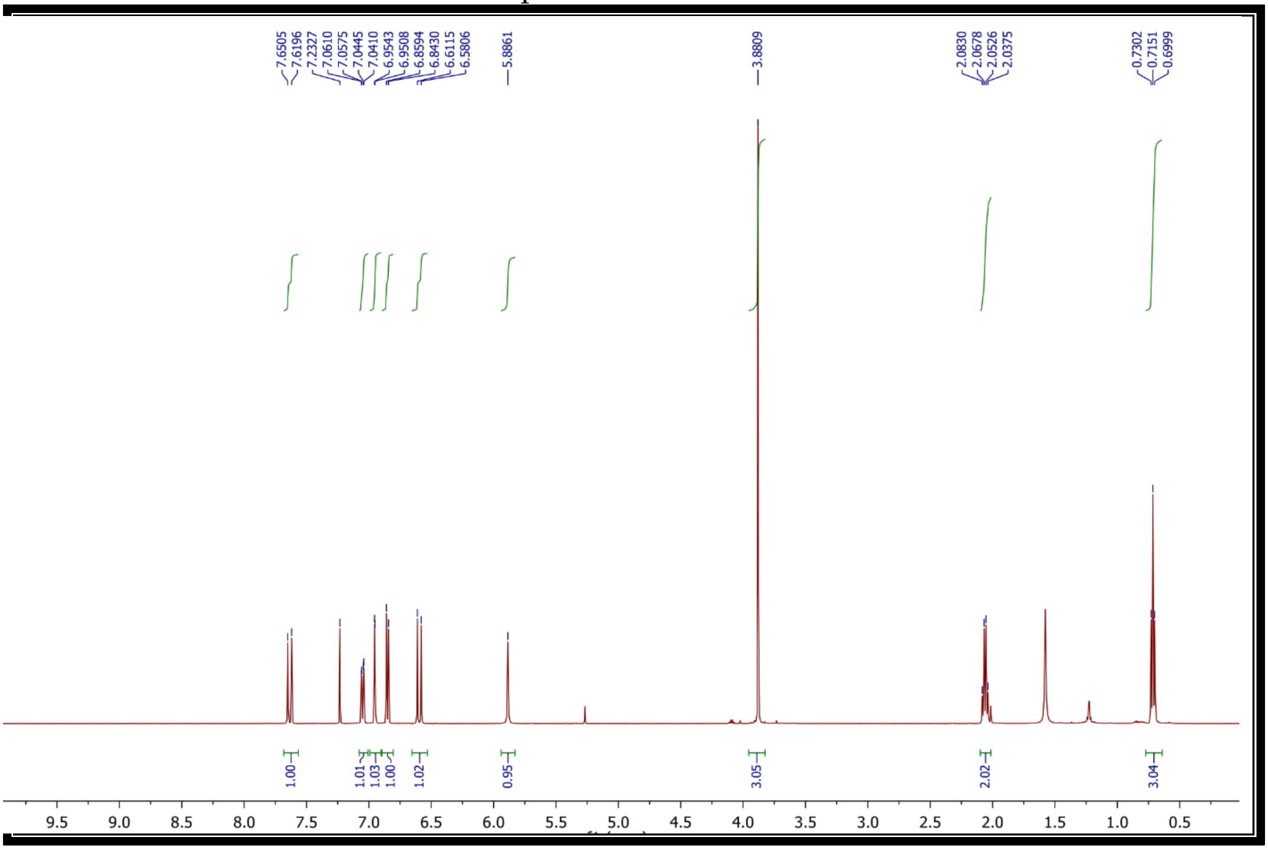

^{1}H spectrum of **3m**

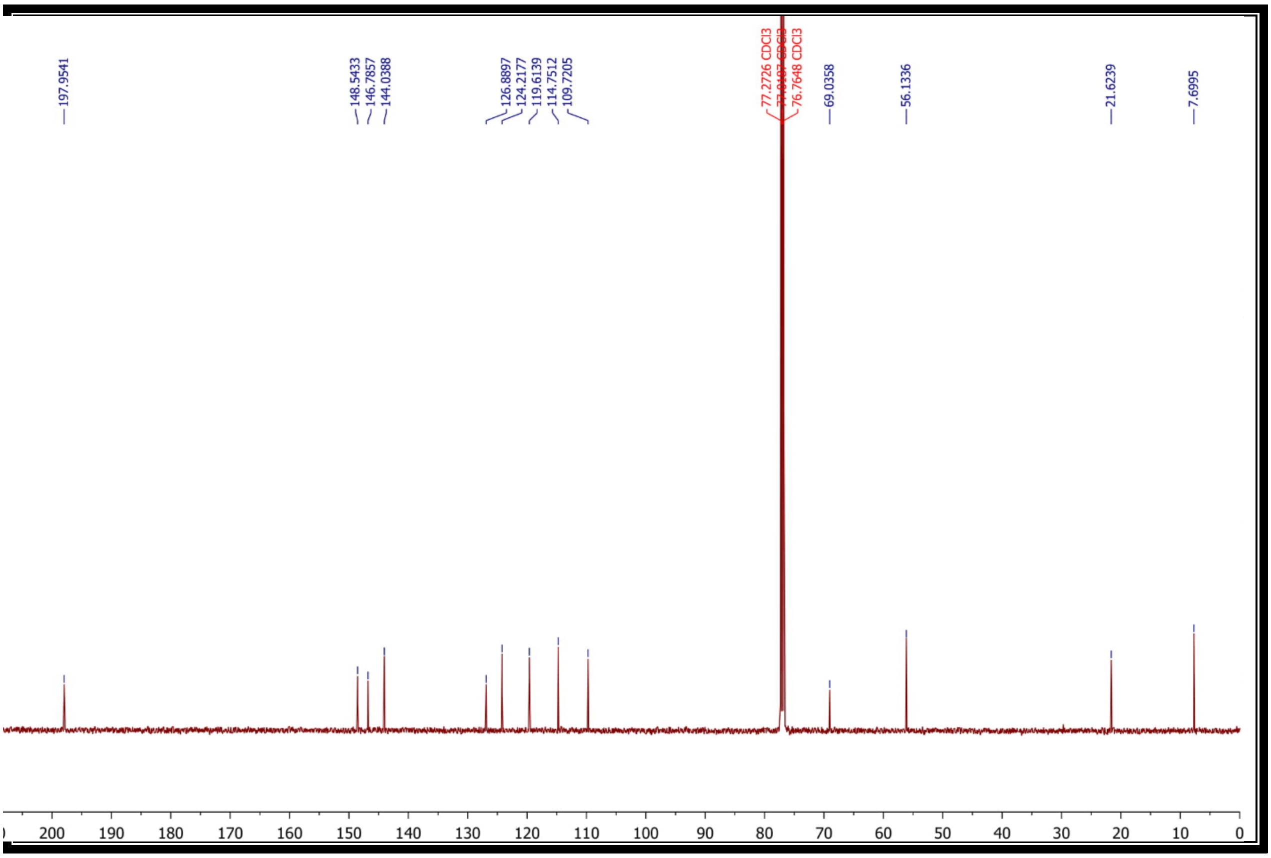

^{13}C spectrum of **3m**

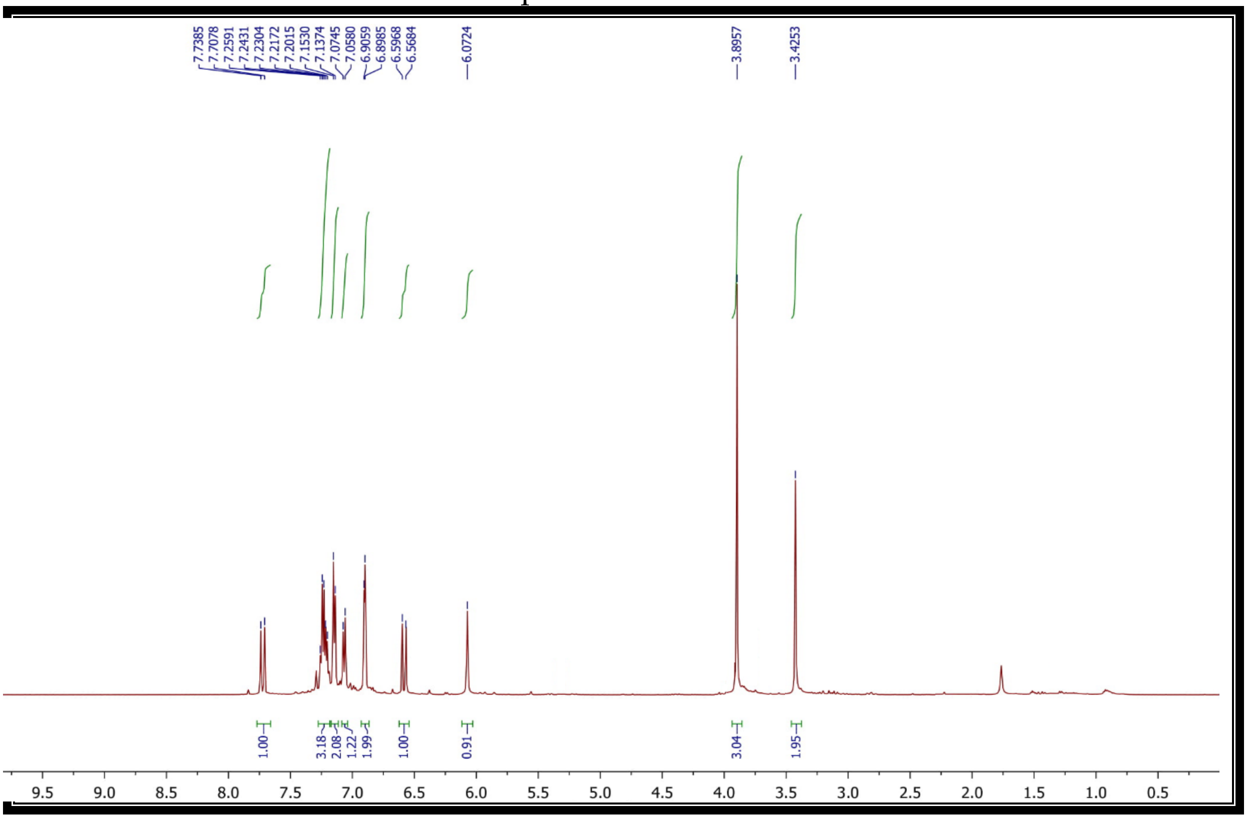

^{1}H spectrum of **4m**

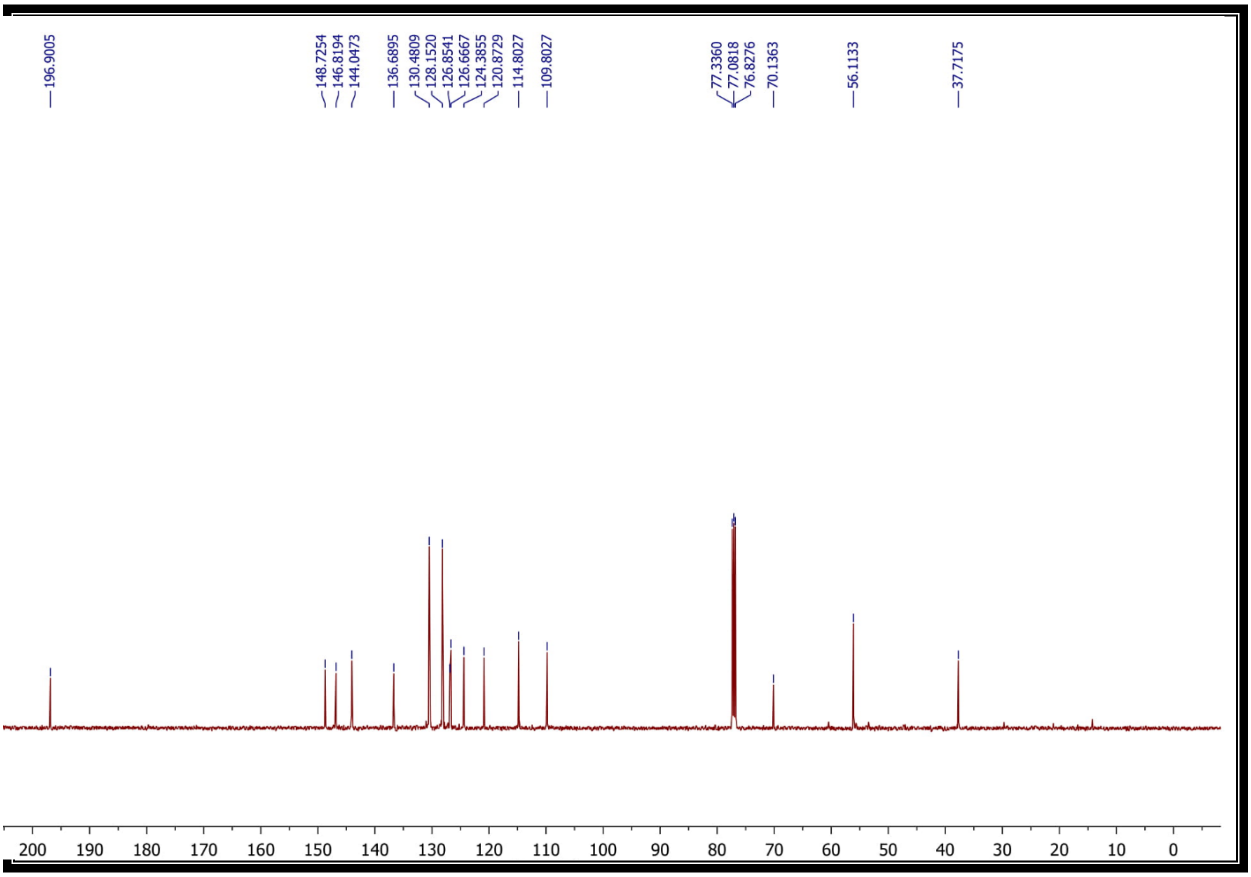

^{13}C spectrum of **4m**

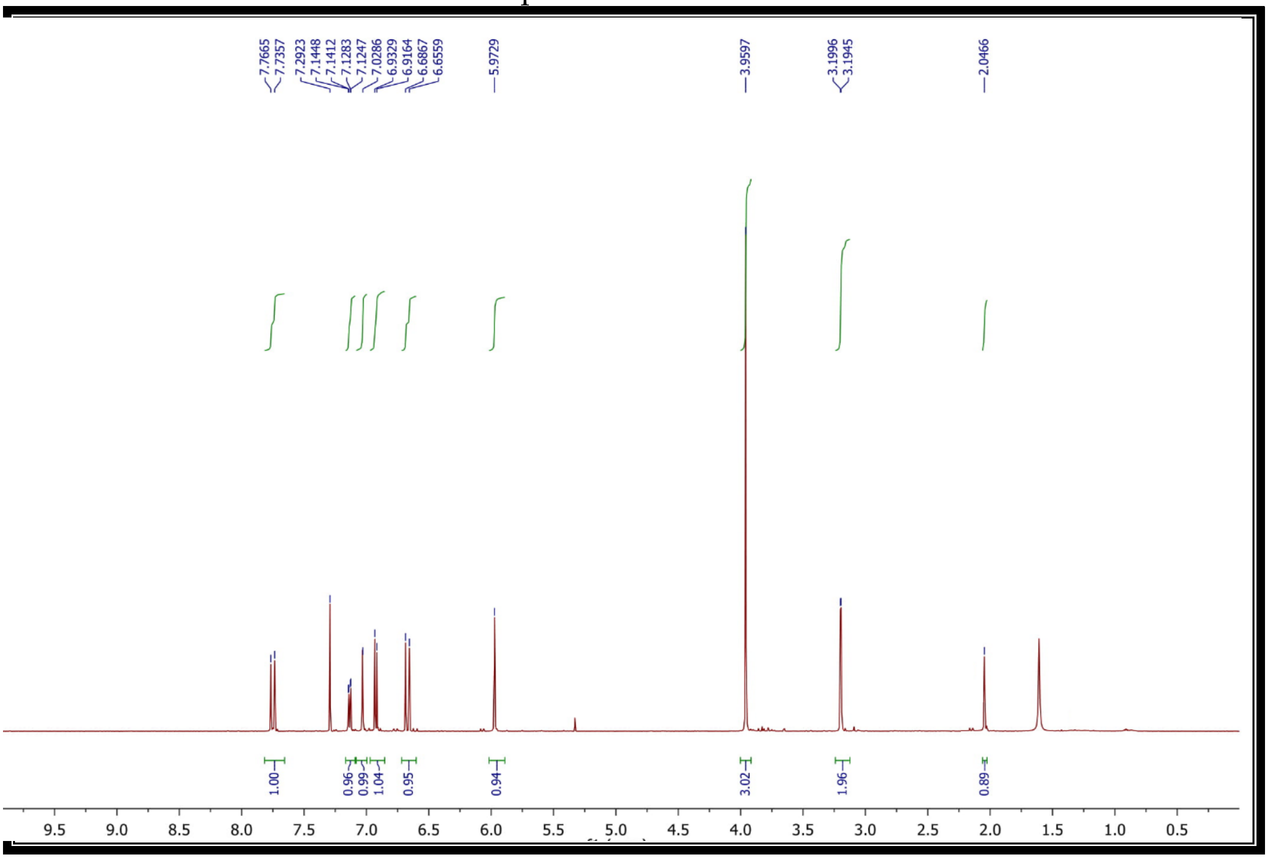

^{1}H spectrum of **5m**

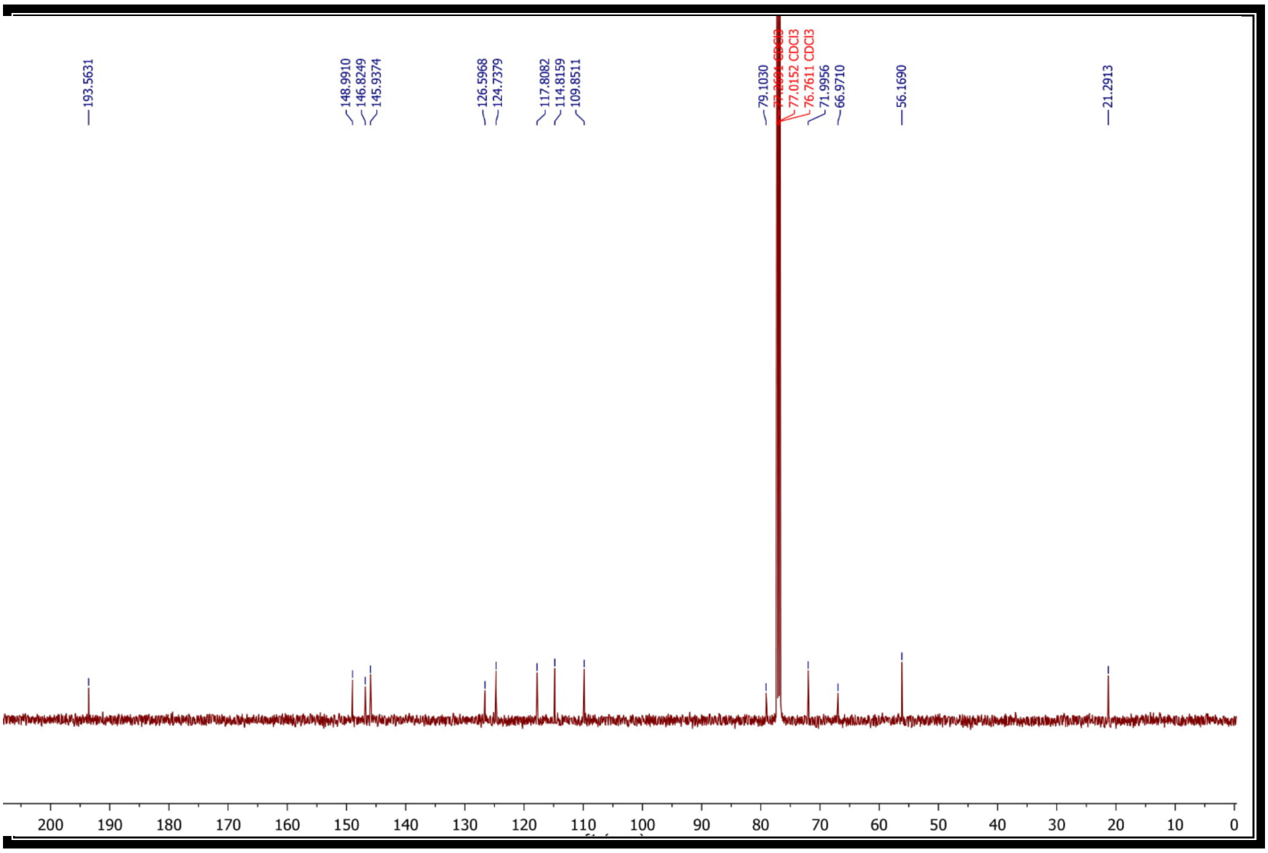

^{13}C spectrum of **5m**

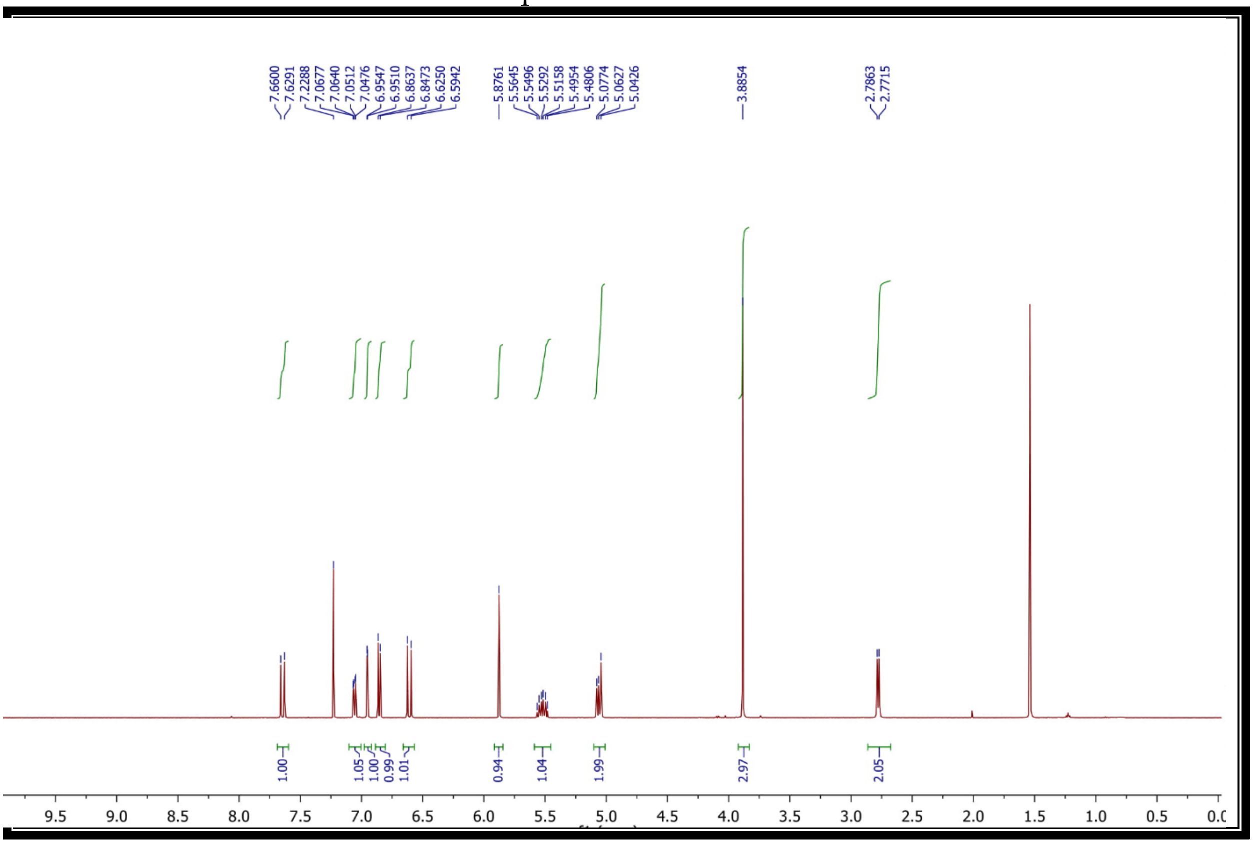

^{1}H spectrum of **6m**

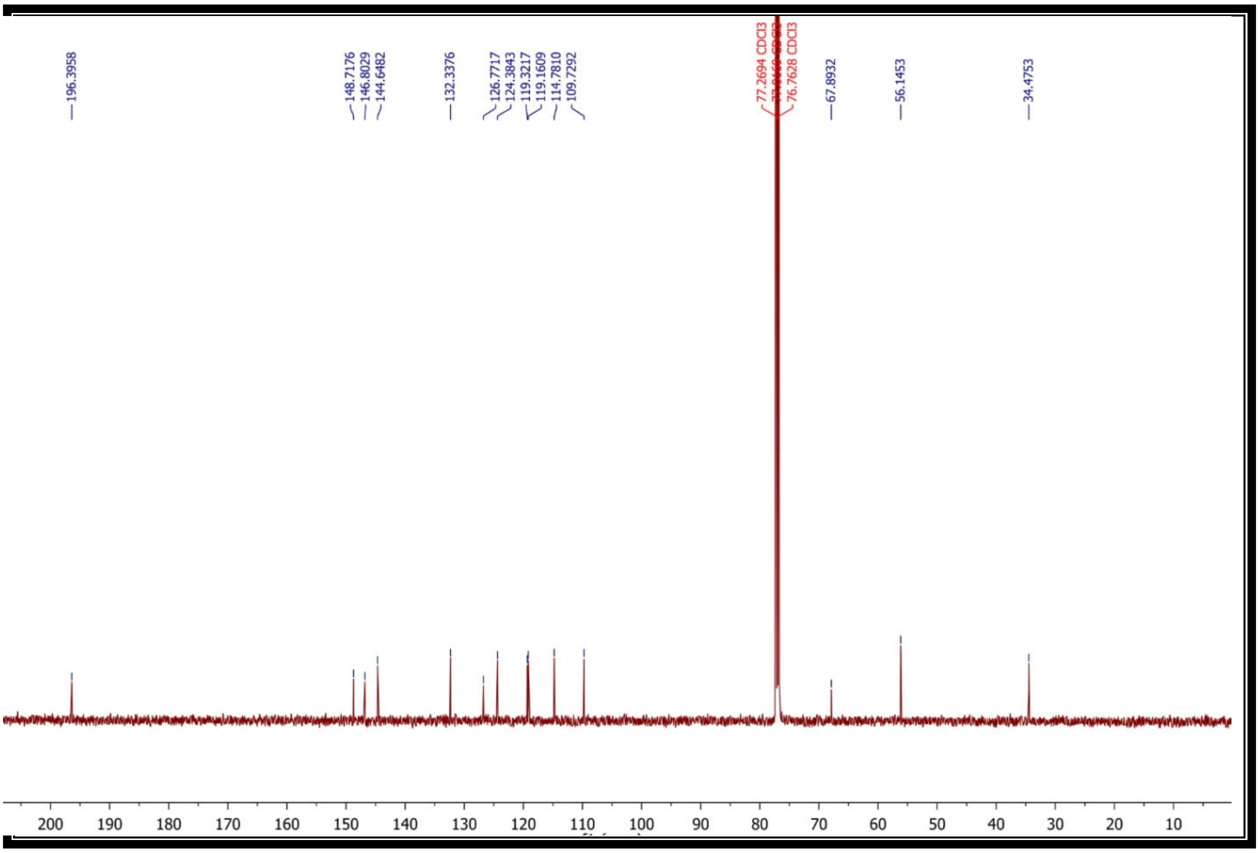

^{13}C spectrum of **6m**

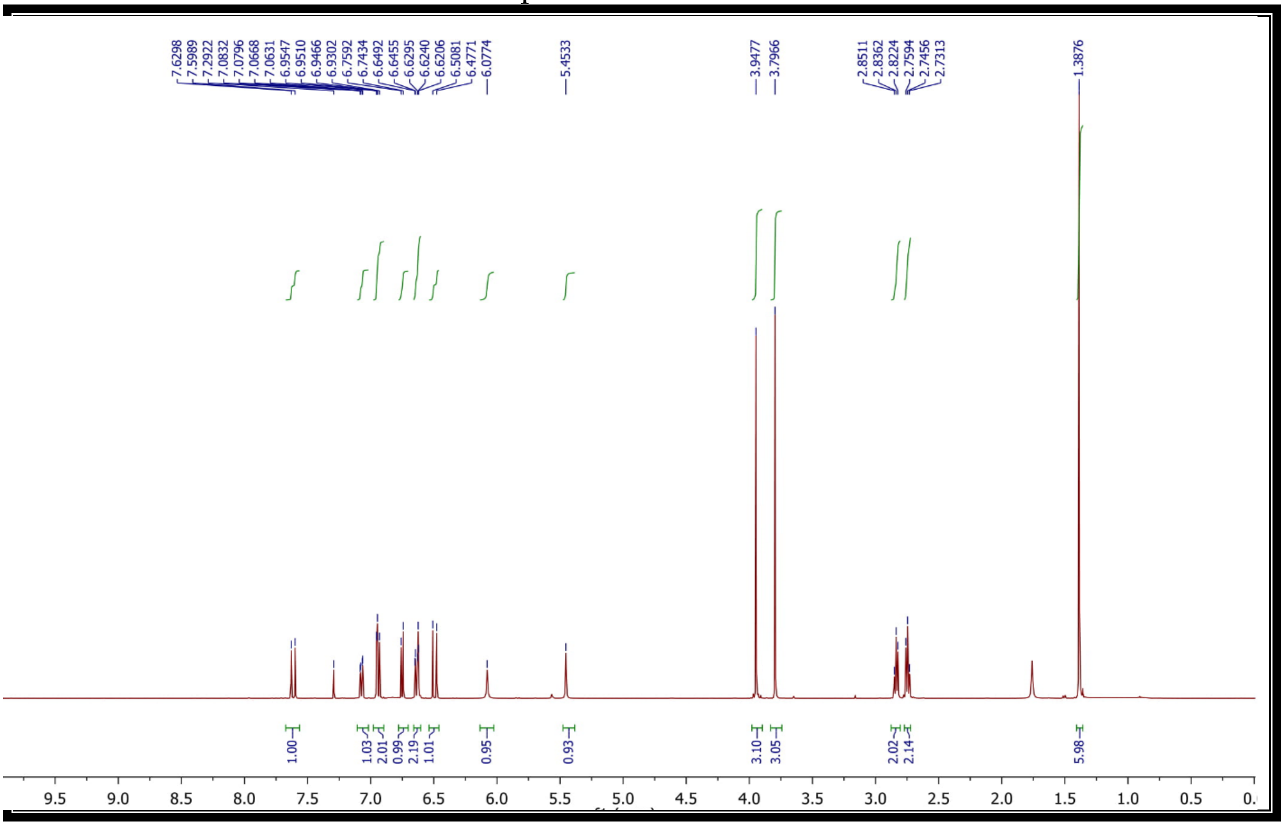

^{1}H spectrum of **18**

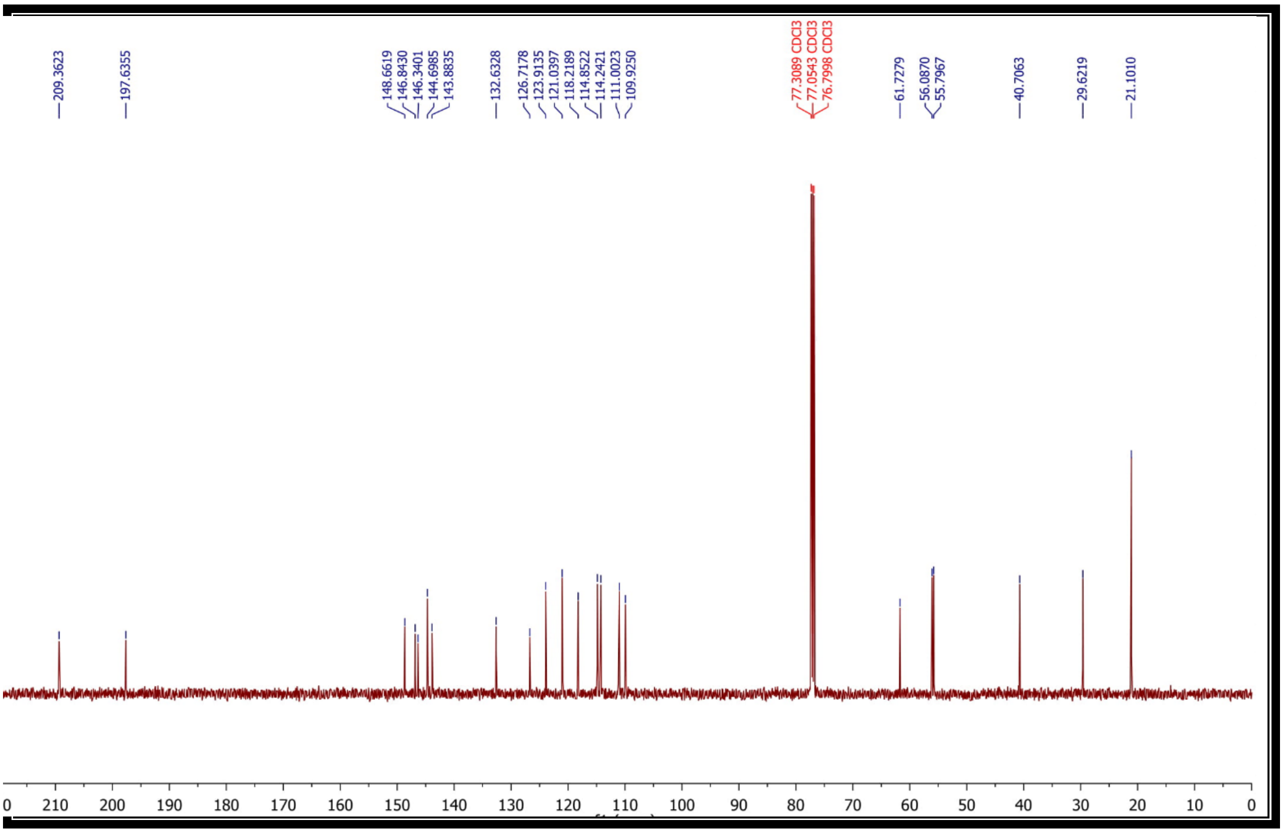

^{13}C spectrum of **18**

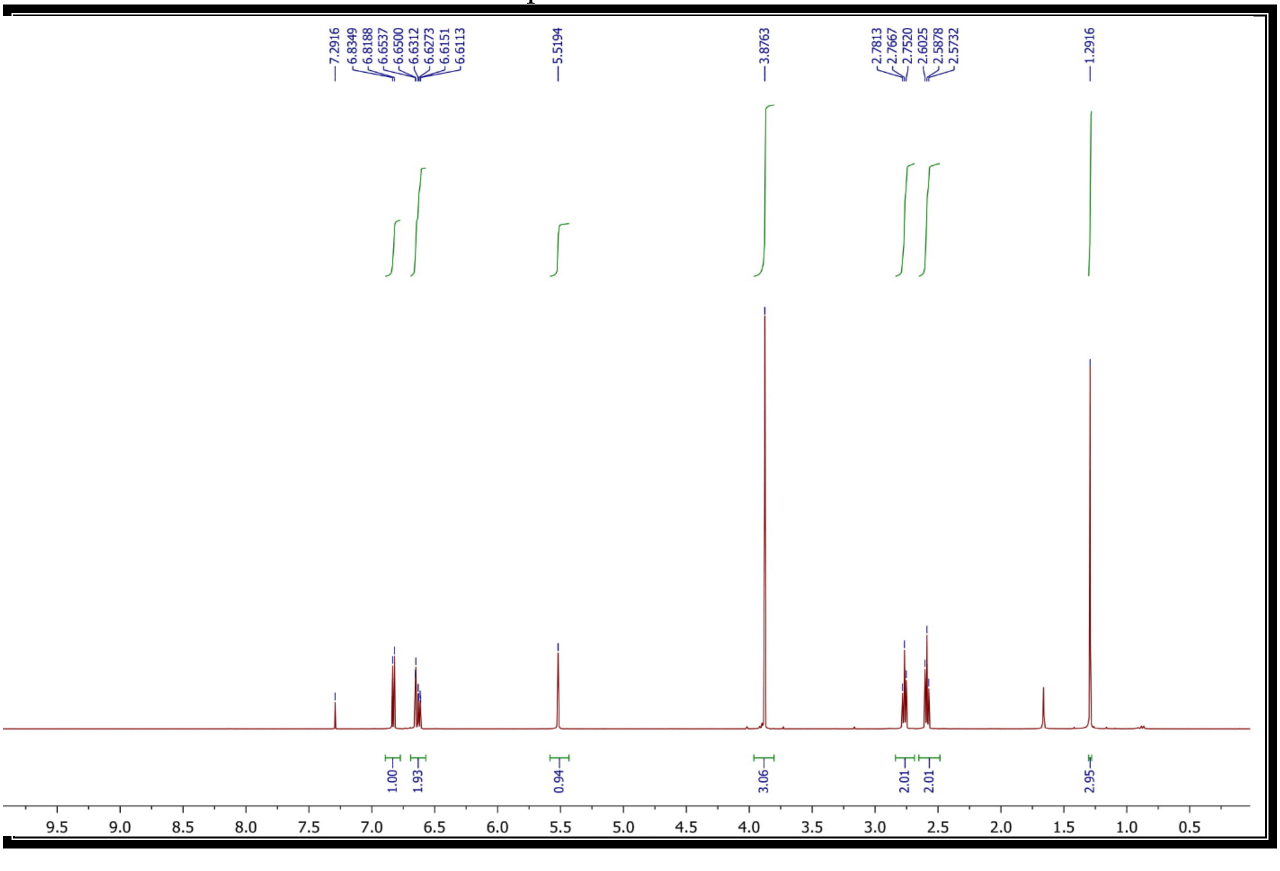

^{1}H spectrum of **19**

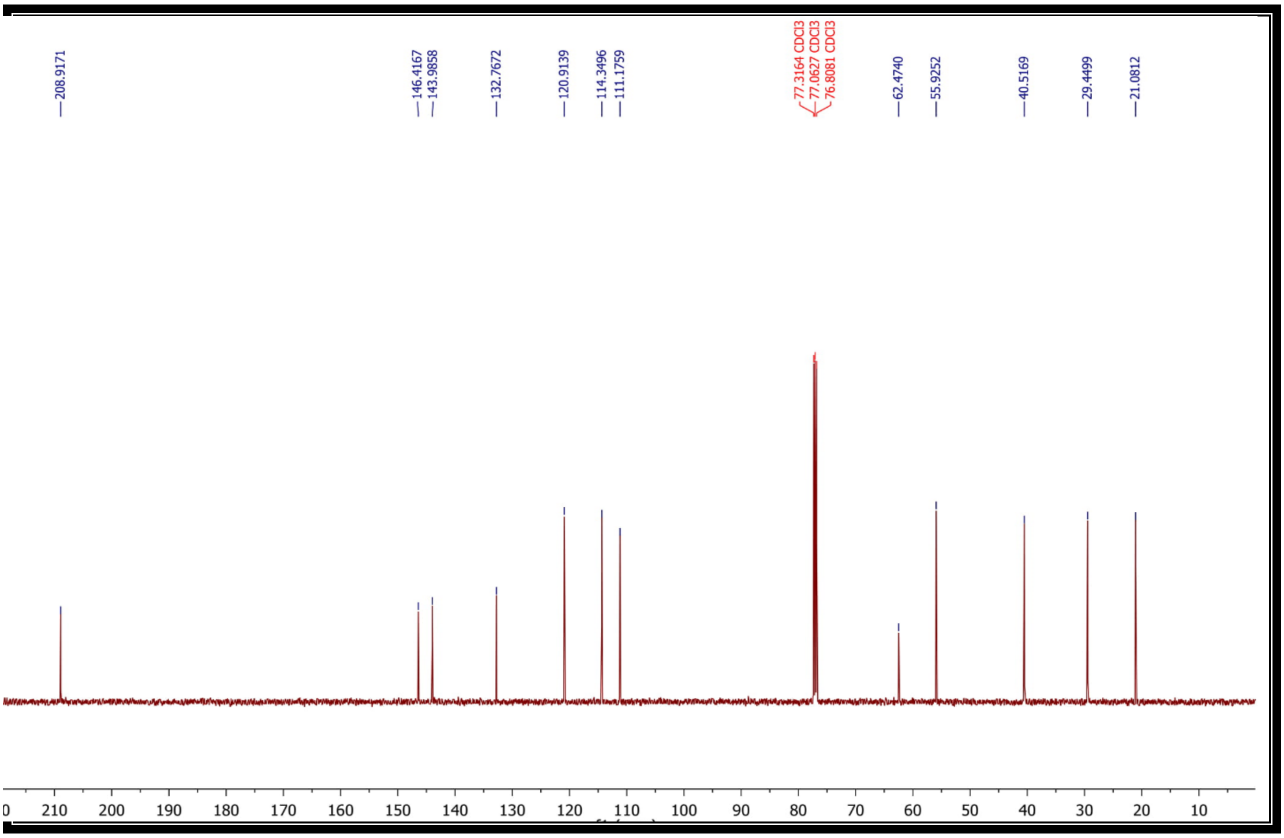

^{13}C spectrum of **19**

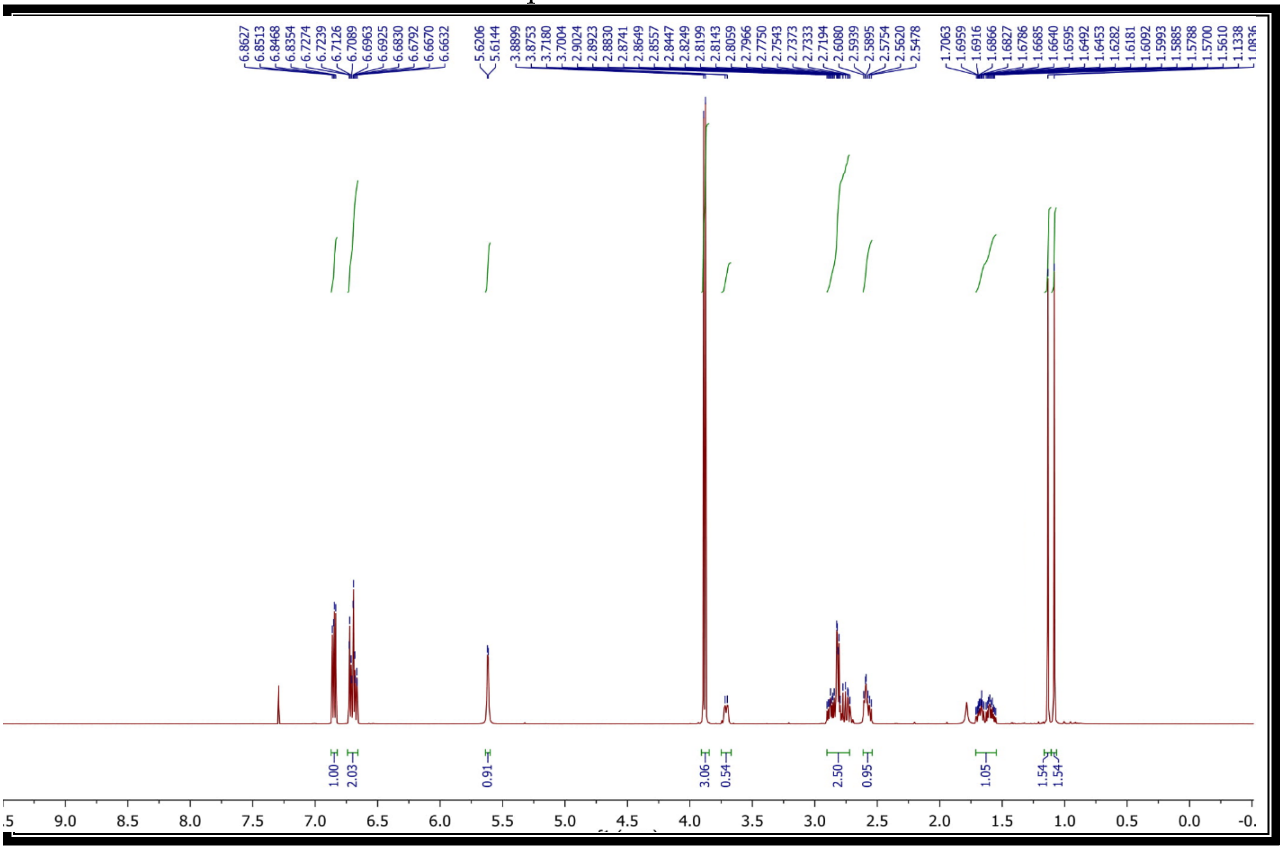

^{1}H spectrum of **20**

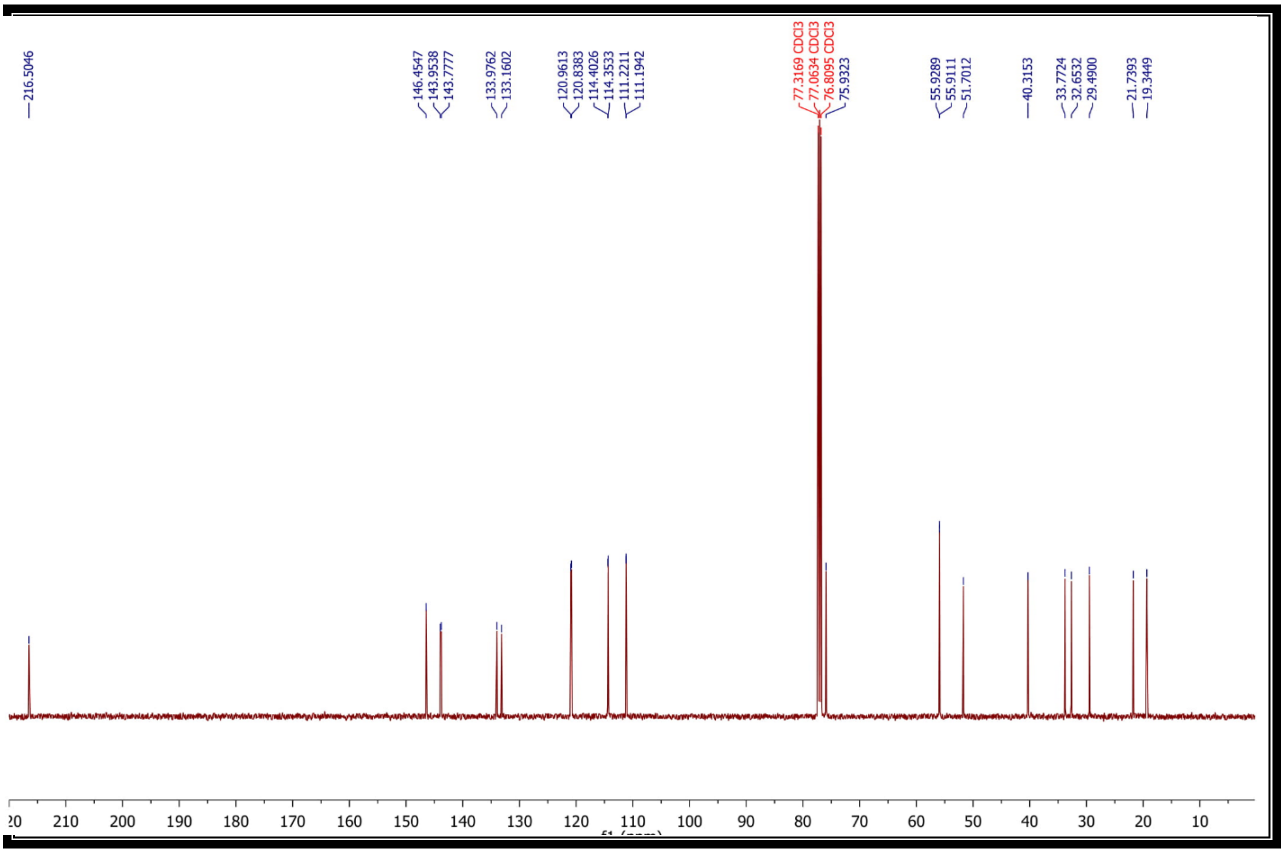

^{13}C spectrum of **20**

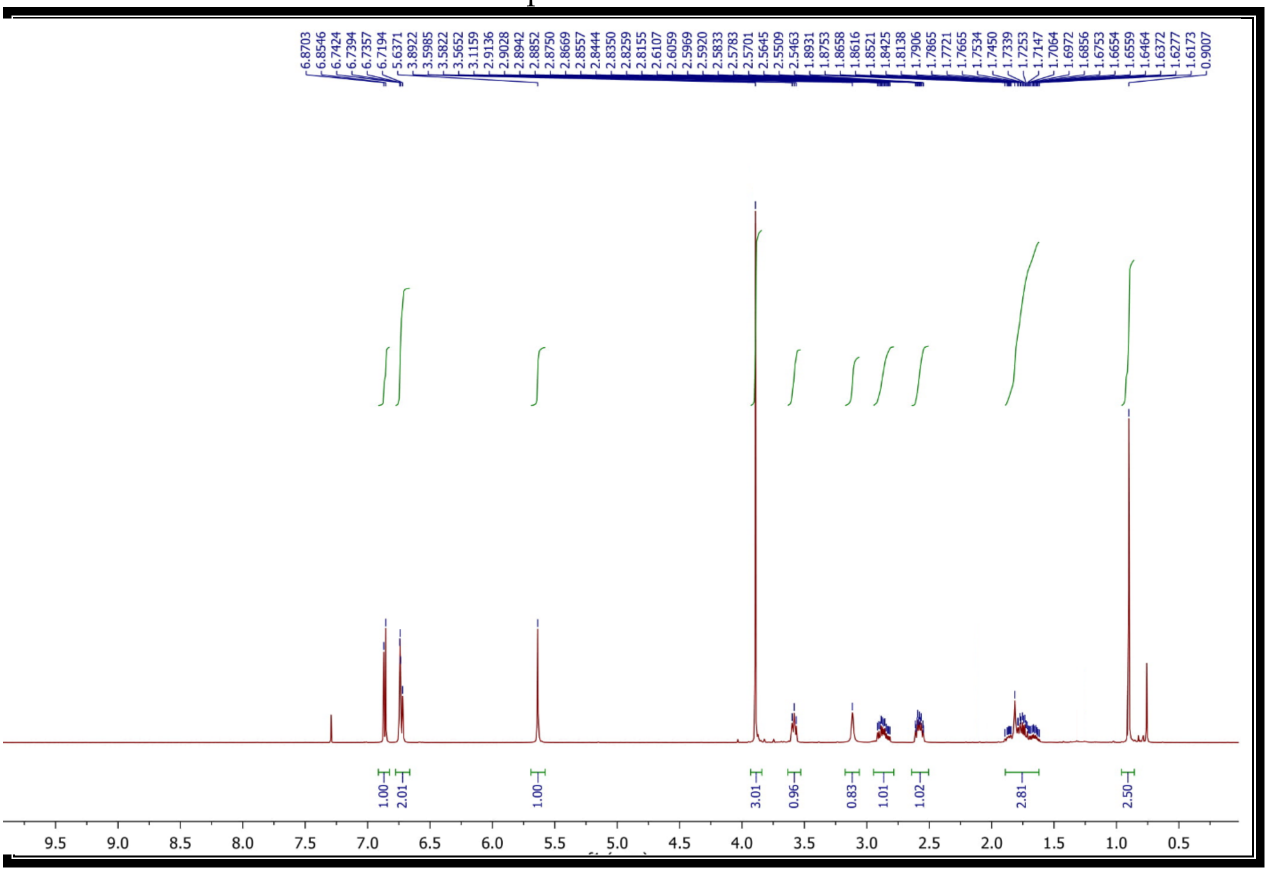

^{1}H spectrum of **21**

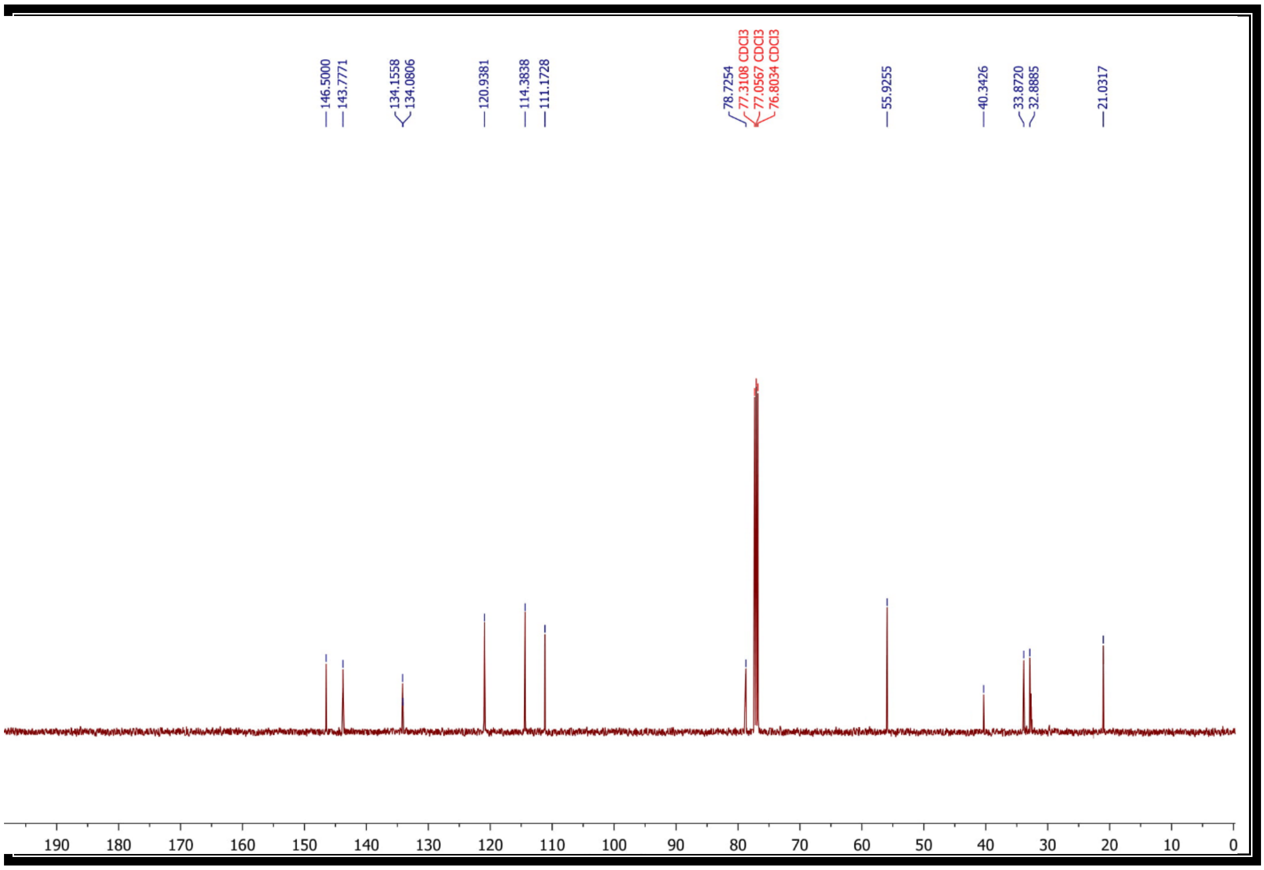

^{13}C spectrum of **21**
